# The Role of KDM2B and EZH2 in Regulating the Stemness in Colorectal Cancer Through the PI3K/AKT Pathway

**DOI:** 10.3389/fonc.2021.637298

**Published:** 2021-03-09

**Authors:** Jaceline Gisliane Pires Sanches, Bo Song, Qingqing Zhang, Xinye Cui, Iddrisu Baba Yabasin, Michael Ntim, Xinlong Li, Jiabei He, Yao Zhang, Jun Mao, Ying Lu, Lianhong Li

**Affiliations:** ^1^Department of Pathology and Forensics, Dalian Medical University, Dalian, China; ^2^Department of General Surgery, First Affiliated Hospital of Dalian Medical University, Dalian, China; ^3^Department of Anesthesiology, First Affiliated Hospital of Dalian Medical University, Dalian, China; ^4^Department of Physiology, Dalian Medical University, Dalian, China; ^5^Department of Ultrasound, Second Affiliated Hospital of Dalian Medical University, Dalian, China; ^6^Teaching Laboratory of Morphology, Dalian Medical University, Dalian, China; ^7^The Key Laboratory of Tumor Stem Cell Research of Liaoning Province, Dalian Medical University, Dalian, China

**Keywords:** KDM2B, EZH2, stemness, colorectal cancer, PI3K/AKT

## Abstract

**Background:** The incidence of colorectal cancer (CRC) has been increasing worldwide in recent years. Targeting cancer stem cells (CSCs) in CRC remains a difficult challenge. KDM2B and EZH2 play important role in the maintenance of CSCs' self-renewal capacity and tumorigenic ability; however, the biological functions of those genes in CRC remain unclear. In this study, we aimed to define the contribution of the expression of KDM2B in the features of CRC and establish the relationship between KDM2B and EZH2 in colorectal CSCs.

**Methods:** The expression of KDM2B and EZH2 in the specimens of CRC and CRC cell lines were analyzed by immunohistochemistry, Western blot, and immunofluorescence. The underlying mechanisms of altered expressions of KDM2B and EZH2 and their impact on the biologic features of CRC and stemness in CRC were investigated.

**Results:** The KDM2B gene was highly expressed in CRC tissues, and its overexpression positively correlated with tumor stages and tumor/node/metastasis (TNM) classification. The downregulation of KDM2B retarded cell proliferation, induced DNA damage, reduced spheroid formation, and decreased CRC stem cell markers: CD44, CD133, and ALDH-1. Moreover, the downregulation of KDM2B decreased the expression of EZH2 and both regulated cell migration, invasion, and stemness in the CRC cell line. Additionally, the interaction between KDM2B and EZH2 significantly increased the components of the PI3K/AKT pathway including AKT and PI3K. The high expression of KDM2B positively correlated with EZH2 in CRC tissues.

**Conclusion:** This study shows that the downregulation of KDM2B and EZH2 can regulate CRC cell stemness, and their interaction may serve as a novel prognostic marker and therapeutic target for patients with CRC.

## Introduction

Colorectal cancer (CRC) is considered one of the most common malignant tumors of the digestive system and is ranked among the most lethal cancers. It is rated as the third highest risk of cancer-related deaths worldwide (1). Although there are effective diagnoses and treatments against CRC, this disease remains a serious threat to millions of people globally.

Cancer stem cells (**CSCs**) are considered a major threat to the treatment response and prognosis of various cancers, including colorectal cancer (2, 3). They are a group of tumor cells with the characteristics of stem cells: self-renewal, infinite proliferation, and the potential of multi-directional differentiation (2, 4). Although CSCs account for a very minor population of cancer, they are firmly related to tumor metastasis, drug resistance, and recurrence after primary treatment (3). Colorectal cancer stem cells (CR-CSCs) share the major biological characteristics of stem cells from other solid tumors. The surface markers (CD44, CD133, CD166, Lgr5, ALDH1, and EpCAM) (5, 6) are efficient in the identification of CR-CSCs. However, studies have reported that the use of CD133 in combination with CD44 seems to be more reliable as target biomarkers (7). The dysregulation of Wnt/β-catenin, Notch, TGF-β, and Hedgehog signaling pathways have been reported in CR-CSCs (8–10). In particular, the activation of the Wnt/β-catenin signaling pathway contributes to the maintenance of cancer stem–like cell properties and drug resistance in CR-CSCs (11).

The oncogenic potential epigenetic regulator lysine–specific demethylase 2B (KDM2B), also known as NDY1, FBXL10, and JHDM1 is a member of the Jumonji C (JmjC) domain-containing histone demethylase (JHDM) family (12, 13). KDM2B regulates gene transcription *via* the demethylation of the dimethyl histone H3 lysine 36 (H3K36me2) and trimethyl histone H3 lysine 4 (H3K4me3) (14). This gene is located on chromosome 12q24.31 and encodes a member of the F-box protein family, which is characterized by ~40 amino acid motifs (15). KDM2B gene expression is associated with several abnormalities, including aniridia, genitourinary anomalies, mental retardation syndrome, and tumors (16). It has been established that the abnormal expression of KDM2B inhibits tumor suppressor genes and promotes oncogene expression, thereby contrib-uting to uncontrolled cell growth and possibly leading to tumorigenesis (17–19). KDM2B controls stem cell self-renewal (20), somatic cell reprogramming and senescence (21), and tumorigenesis (13). KMD2B is also highly expressed in embryonic stem cells, hematopoietic stem cells, leukemia, and solid cancers (18, 22, 23). Staberg et al. (24) demonstrated that KDM2B plays an important role in glioblastoma (GBM), where it critically maintains glioblastoma cell survival, genome integrity, stem-like tumor populations, and maintenance of glioblastoma stem-like cell (GSC) pools. Although studies have demonstrated that KMD2B regulates cancer stemness, the expression roles and regulatory mechanism of KDM2B in CR-CSCs have not been studied.

Recently, studies have shown that the enhancer of zeste homolog 2 (EZH2) is an important regulator of the development of cancer development and progression (25–27). EZH2 is a component of PRC2 that mediates methylation of histone H3 methylated Lys 27 (H3K27) and functions in the maintenance of embryonic stem cell pluripotency and plasticity (25, 28). In various cancers, targeting these genes (EZH1 and EZH2) has tumor-suppressive functions affecting tumor cell proliferation, invasion, and metastasis (26, 27). EZH2 expression is regulated by various oncogenic transcription factors and cancer-associated non-coding RNA that are critical for cell proliferation, tumorigenesis, and stem cell maintenance (29, 30). Studies have reported that EZH2 inhibition and knockdown dramatically decrease the tumorigenicity of CSCs (30, 31). Previously, Cheng et al. (31) found that EZH2 promotes CRC stem-like cell expansion by activating p21 cip1 -Wnt/β-catenin signaling, supporting the hypothesis that EZH2 may serve as a novel CSC marker and a potential target for cancer therapy. Moreover, EZH2 gene expression has been reported to be regulated by KDM2B in several abnormalities (23, 32). KDM2B and EZH2 both seem to play an important role in the maintenance of the self-renewal capacity and tumorigenic ability of CSCs. However, the correlation between the expressions of KDM2B and EZH2 and CRC stemness remains unclear.

The impact of the PI3K/AKT signaling pathway in the development of cancer and progression is well-documented. This signaling is crucial in cancer because it advances cell growth and survival (33, 34). Apart from its role in a solid tumor, the PI3K/Akt pathway plays an important role in CSCs. A previous study reported that the PI3K/Akt pathway plays an important role in the sphere formation and growth of the colon CSCs, which represent properties of stemness and proliferation of CSCs (35). Emphasizing the interest in this particular signaling pathway remains a chance and a challenge for cancer therapy.

In the present study, we aimed to investigate the functional role of KDM2B in CRC and the relationship between KDM2B and EZH2 in CR-CSCs. We found that the endogenous level of KDM2B was high in CRC tissues compared with normal tissues, and its expression was strongly related to the clinical stage and TNM stage. The downregulation of KDM2B decreased the viability of CRCs and induced DNA damage, concluding that KDM2B might act as an oncogene. The knockdown of KDM2B inhibited the spheroid formation of CRC cells and decreased the expression of surface markers of CRC-CSCs (CD133, CD44, and ALDH-1). Additionally, the downregulation of endogenous KDM2B decreased the expression of EZH2 and activated the PI3K/AKT pathway. KDM2B and EZH2 were indispensable for the maintenance of CSCs *in vitro* by regulating the PI3K/AKT signaling pathway. The knockdown of KDM2B and EZH2 reduced migration in CRC cells. The expression of EZH2 is positively related to KDM2B in tissues of CRC. KDM2B might be a potential therapeutic target for CRC.

## Materials and Methods

### Human Colorectal Cancer—Clinical Specimens

The tissue microarrays (TMAs), including 150 paraffin-embedded primary CRC tissues, were obtained from the Shanghai Outdo Biotech Company (Shanghai, China). The human tissue samples were used according to the regulations of the Medical Ethics Committee of the Second Affiliated Hospital of Dalian Medical University. The TMAs include 75 cases of tumor tissues and 75 cases of adjacent normal tissues (HColA150CS02). The clinical diagnosis and demographic information, including age, sex, tumor size, clinical staging, and tumor/node/metastasis (TNM) staging classification were provided by the company and summarized in **Table 2**.

### Cell Culture

Human normal colorectal epithelial cells (CCD 841CoN) and colorectal cancer cell lines (LOVO, ROK) were obtained from the Laboratory of Pathology at Dalian, Medical University, whereas HT-29 was purchased from the Cell Research company (Shanghai, China) and DLD-1 was from BeNa Culture Collection (BNCC, Jiangsu, China). CCD 841CoN and LOVO cells were cultured in RPMI-1640 medium (Gibco, USA) and HT-29, DLD-1, and ROK cells were cultured in Dulbecco's Modified Eagle Medium (DMEM) /high glucose (Hyclone, Logan, UT, USA). The composite culture mediums were supplemented with 10% fetal bovine serum (FBS), 50 IU/ml, and 50 μg/ml of penicillin and streptomycin and incubated at 37°C in a humidified incubator with 5% CO_2_.

### Immunohistochemical Analysis

The expression of KDM2B and EZH2 proteins in the specimens with CRC was analyzed by immunohistochemistry using the TMA slide. The tissues were deparaffinized, rehydrated, and treated with citric acid buffer (pH 6.0) at 95–100°C, 10 min for antigen retrieval. Endogenous peroxidase activity was blocked by incubation in 3% H_2_O_2_ for 20 min. Tissues were then incubated with primary polyclonal anti-JHDM1B antibody (1:500; MILLIPORE China) and polyclonal anti-rabbit EZH2 antibody (1:300; Proteintech, China) in a humidified chamber overnight at 4°C. The following day the slides were washed with phosphate-buffered saline (PBS) and incubated with secondary antibody (Santa Cruz Biotechnology, USA) for 30 min. After washing, the detection was determined by a non-biotin horseradish peroxidase detection system and DAB substrate (Dako, USA). The tissues were counterstained by hematoxylin (Tianjin Fuyu Fine Chemical Co. Ltd, Tianjin, China) and mounted in Distyrene, Plasticiser, Xylene (DPX) (Tianjin Fuyu Fine Chemical Co. Ltd, Tianjin, China). Immunohistochemistry results of the expression of KDM2B in CRC were observed and evaluated by two pathologists independently. Both pathologists were blinded to the sample type prior to evaluating samples. The expressions of KDM2B and EZH2 were evaluated using a semi-quantitative immunohistochemical score (0–12points) depending on the intensity (0–3 points) and proportion (0–4 points) (36). The staining intensity was scored as follows: 0 = negative staining, 1 = weak staining, 2 = moderate staining, and 3 = strong staining. The proportion of positive staining was scored as follows: no staining was 0, <25% was 1, 25–50% was 2, 51–75% was 3, and > 75% was 4. The final staining scores were calculated by multiplying the intensity score by the extent of positive cells staining, which yielded a range from 0 to 12 points.

### siRNA, shRNA, and Antibodies

The design and synthesis of siRNAs were completed by RiboBio (Guangzhou, China). The lentivirus shRNA of KDM2B-knockdown and shRNA of EZH2-knockdown constructs were purchased from GenePharma (Shanghai, China). The negative control siRNA (si-NC) and shRNAs (NC) were provided by corresponding companies. Antibodies were purchased from different companies including, KDM2B (MILLIPORE, China), EZH2, CD133, CD44, ALDH1, P21, P27, β-tubulin (ProteinTech, China), Cyclin D1 (Boster, China), and antibodies against PI3K, pPI3K, AKT, and pAKT, were purchased from Wanleibio and Invitrogen (China, USA). Secondary antibodies were purchased from Santa Cruz Biotechnology (Dallas, TX, USA).

### Cell Transfection and Lentivirus Infection

Twenty-four hours prior to transfection, HT-29 and DLD-1 cells were seeded into 6-well plates at a density of 5 × 10^5^ cells per well. The cells were then transfected with KDM2B siRNAs (siKDM2B#1 and siKDM2B#2) or negative control (NC) siRNA. The siRNAs were transfected using Lipofectamine 2000 (Invitrogen, USA). Lentivirus vectors, including short hairpin RNA against KDM2B (KDM2B-Homo-1621) and EZH2 (EZH2-homo-488), and the negative control (LV-N) were added to infect the cells for 72 h. Positive cells were screened using 1 mM of puromycin (Sigma-Aldrich, St. Louis, MO) for 2 weeks. Cells expressing Green Fluorescent Protein (GFP) were observed under fluorescence microscopy (Olympus IX73; Olympus, Tokyo, Japan). The expression of KDM2B in the infected cells was confirmed by Western blotting and qRT-PCR.

### RNA Extraction and Real-Time qRT-PCR

Total RNA was extracted from transfected HT-29 and DLD-1 cells using TRIzol reagent (Invitrogen, Carlsbad, CA), and cDNA was synthesized with All-in-one First-Strand cDNA Synthesis SuperMix kit (Transgen, China). The RT-qPCR amplification for KDM2B mRNA was analyzed by Agilent MX3000P, and an SYBR Premix EX Tag Master mixture kit (Transgen, China) according to the instructions from the manufacturer. The relative expression levels of mRNA were evaluated using the 2^−ΔΔCt^ method after normalizing to GAPDH. All qRT-PCR experiments were done in triplicate. The sequences of the primers used for the targeted gene in this study are shown in Supplementary Table 1.

### Cell Proliferation and Colony Formation Assays

To further assess the growth potential of KDM2B in CRC cells, cell proliferation and colony formation assays were employed. For the proliferation assay, 24 h after transfection, HT-29 and DLD-1 cells were seeded at a density of 3 × 10^3^ cells per well in 96-well microplates in triplicates, and proliferative cells were stained with CKK-8 (Sigma-Aldrich, USA) and measured at 0, 24, 48, and 72 h. Two hours after adding 10 μl of CCK-8 reagent, the absorbance was measured at 450 nm using the Multiskan Go spectrometer (Thermo Fisher, USA). For the cell colony formation assay, 24 h post-transfection the cells mentioned above were harvested and seeded in 6-well plates at a density of 500 cells per well and incubated for 6 days for the formation of colonies. The colonies were fixed and stained with 1% crystal violet for 20 min and then counted.

### Comet Assay

Comet assay was conducted to determine whether the downregulation of endogenous KDM2B could be attributed to DNA damage in CRC cells. The assay was carried out according to the instructions from the manufacturer. Briefly, 48 h after transfection HT-29 and DLD-1 cells were harvested and redistributed in 1 × PBS to a density of 1 × 10^5^ per 1 ml. The control group and siRNAs groups (siKDM2B#1 and siKDM2B#2) were mixed with molten low melting agarose gel (LMA gel) at a ratio of 1:10 (50 μl of cells in 1 × PBS to 1 × 10^5^/ml 500 μl of LMA agarose at 37°C), then low melting agarose gel containing cells were sandwiched with normal melting agarose (NMA) gel on slices and placed in the refrigerator for 10 min at 4°C. Slides were then immersed in lysis buffer at 4°C overnight. Twenty-four hours after incubation in lysis buffer, the residual buffer on the slides was removed and submerged in a newly constituted alkaline unwinding solution (pH > 13) for 20 min at 4°C in a refrigerator. After 20 min of incubation, slides were placed in an electrophoresis tray containing alkaline solution and covered with a slide tray overlay. The power supply was set to 25 V and the voltage was applied for 30 min. After 30 min, the excess electrophoresis solution was drained and carefully immersed thrice in 0.4 mmol Tris-HCL (pH 7.5) for 10 min each. Following incubation, slides were dried at 37°C 10–15 min and were then incubated in propidium iodide (PI) for 10 min in darkness. Slides were viewed with a fluorescence microscope (Olympus IX73, Olympus Corporation, Tokyo, Japan). Nuclear DNA and migrating DNA (comet tail) labeled with PI appeared red under the fluorescence microscope. Cells were examined using CaspLab software—the comet assay software project used to measure the level of DNA damage in single-cell gel electrophoresis. The assay was conducted three consecutive times.

### Sphere Formation Assay

The sphere formation assay was executed in ultra-low attachment 6-well plates (Corning, USA). At a density of 1,000 cells/ well, cells were grown in DMEM/F12 supplemented with B27 (1:50, Invitrogen), 20 ng/ml human recombinant EGF (Sigma-Aldrich, USA), and 20 ng/ ml bFGF (Sigma-Aldrich, USA) for 7 and 14 days. Spheres larger than <50 μm in diameter were counted at 20 × and 40 × magnification under an inverted microscope (Olympus Corporation, Tokyo, Japan). For Western blotting, spheres were maintained and collected after 20 days.

### Cytoplasmic and Nuclear Protein Fractionation

The preparation of cytoplasmic and nuclear protein fractions was performed according to the manual from the manufacturer using the cytoplasmic and nuclear extraction reagent kits (Thermo Scientific, USA). In brief, cells were harvested from culture plates and resuspended in cytoplasmic extraction buffer, and incubated for 10 min. The cells were then Dounce-homogenized and centrifuged at 1,000 × g for 5 min at 4°C. The nuclei in the pellet were then resuspended in nuclear extraction buffer and isolated *via* centrifugation at 1,500 × g for 30 min. Both supernatants (cytoplasmic fraction and nuclear fraction) were stored at −20°C until use.

### Western Blotting

Cells were collected by centrifugation and total protein was harvested with a protein extraction cocktail containing lysis and 1 × RIPA buffers (Sigma Chemicals, St. Louis, MO, USA). Easy II Protein Quantitative Kit was used to estimate the protein concentration, and equal concentrations were loaded for SDS-PAGE and separated by electrophoresis. After running SDS-PAGE, proteins were transferred to PVDF membranes (Millipore, Billerica, MA, USA) and blocked for 1 h with 5% non-fat milk dissolved in TBST and then incubated overnight at 4°C with the specific antibodies. The specific antibodies were diluted as follows: KDM2B (1:1,000), EZH2 (1:1,000), CD133 (1:500), CD44 (1:500), ALDH1(1:1,000), P21 (1:500), Cyclin D (1:500), P27 (1:1,000), PI3K (1:1,000), pPI3K (1:500), AKT (1:500), pAKT (1:1,000), and β-tubulin (loading control) (1:1,000). The following day, the incubated membranes were washed and incubated with anti-IgG secondary antibodies (1:16,000 in TBST) for 1 h at 37°C. The protein band images were captured with ODYSSEY infrared imaging system (version 3.0 software, LI-COR Biosciences).

### Immunofluorescence Staining

The immunofluorescence was carried out following protocol. In summary, cells were grown to 3 × 10^3^ in 24-well plates at 37°C and fixed with 4% paraformaldehyde for 20 min. Next, cells were permeabilized with PBS containing 0.5% Triton X-100 for 10 min at room temperature. After blocking using 3% BSA reagent, cells were incubated with primary antibodies for KDM2B and EZH2 (ProteinTech, China) overnight at 4°C. Subsequently, FITC conjugated secondary antibody (Invitrogen) was added and incubated for 1 h, then stained with 1 μg/ml of DAPI (Beyotime, China). Images of samples were visualized and captured using a fluorescence microscope (Olympus IX73; Olympus, Tokyo, Japan).

### Magnetic Sorting-Based Separation

CD133^+^ and CD44^+^ cells are known to express a high variety of stemness genes. Furthermore, the combined analysis of putative co-CSC markers CD133 and CD44 seems to be more reliable as target biomarkers of low- and high-risk cases of CRC, as compared to single-marker analyses (37). To delineate the expressions of KDM2B and EZH2 on those co-CSC markers, CD133^+^/ CD44^+^ or CD133^−^/ CD44^−^ cells were isolated from HT-29 cells and separated using a magnetic column included in the MicroBead kit according to the protocol from the manufacturer CD133 MicroBead kit (cat. no. 130-091-895) and CD44 MicroBead kits (cat. no. 130-095-194); both were purchased from Miltenyi Biotec. Averages of 1 × 10^7^ HT-29 cells were centrifuged, and the supernatant was discarded. CD133 microbeads were added and incubated with the cells. Prior to sorting, the column was placed in a magnetic field and rinsed, and the cells were then loaded onto the column (LD Columns, LS Columns, cat. no. 130-042-901, and cat. no. 130-042-401). The acquired CD133^−^ or CD133^+^ cells were incubated with CD44 microbeads (Miltenyi Biotec) and stained with antibodies at 4°C for 15 min. Cells were again washed and magnetically separated.

### Wound Healing Assay

To further probe the effect of KDM2B and EZH2 on the migratory ability of CRC cells, a scratch wound healing assay was performed. An average of 2 × 10^5^ transfected cells was seeded in 6-well plates and incubated at 37°C in 5% CO_2_. When cellular density reached nearly 100%, cells were gently wounded with a 200-μl pipette tip, and debris was washed off three consecutive times with PBS. Cells were then cultured in the serum-free medium for 24, 48, and 72 h. The wound closure area was micrographed at 0, 12, 24, and 48 h. All assays were performed in triplicate.

### Cell Migration and Invasion

A seeding density of 1 × 10^5^ cells in triplicate was placed in the upper chamber of the 24 well plates (pore size 8 μm, Corning, USA) containing 200 μl of serum-free DMEM medium. For invasion assay, the base of the upper chambers was pretreated with extracellular matrigel (BD, Bioscience, San Jose, CA, USA) to serve as an artificial membrane. The lower chambers were filled with 700 μl complete medium to serve as a chemoattractant. A medium supplemented with 20% serum was added into the lower chamber and incubated at 5% CO_2_ at 37°C in a humidified incubator. After 48 h of seeding, cotton swabs were used to clean the upper inserts. The cells that penetrated on the other side of the membrane were fixed in methanol for 10 min and stained with 1% crystal violet. A randomly selected area was counted under the microscope (Olympus IX73; Olympus Corporation, Japan).

### Statistical Analysis

Statistical analysis was performed with SPSS version 15.0 and Graph pad version 8 (Graph Pad Software. Inc., USA). Differences between the two groups were evaluated by Student *t*-test. One-way ANOVA was used when comparing multiple groups. Clinical data were analyzed using the chi-square test. The data were presented as mean ± SD and statistical significance was set at *p* < 0.05.

## Results

### The Overexpression of KDM2B Is Directly Related to Pathologic Features in CRC

**Figure 1:**
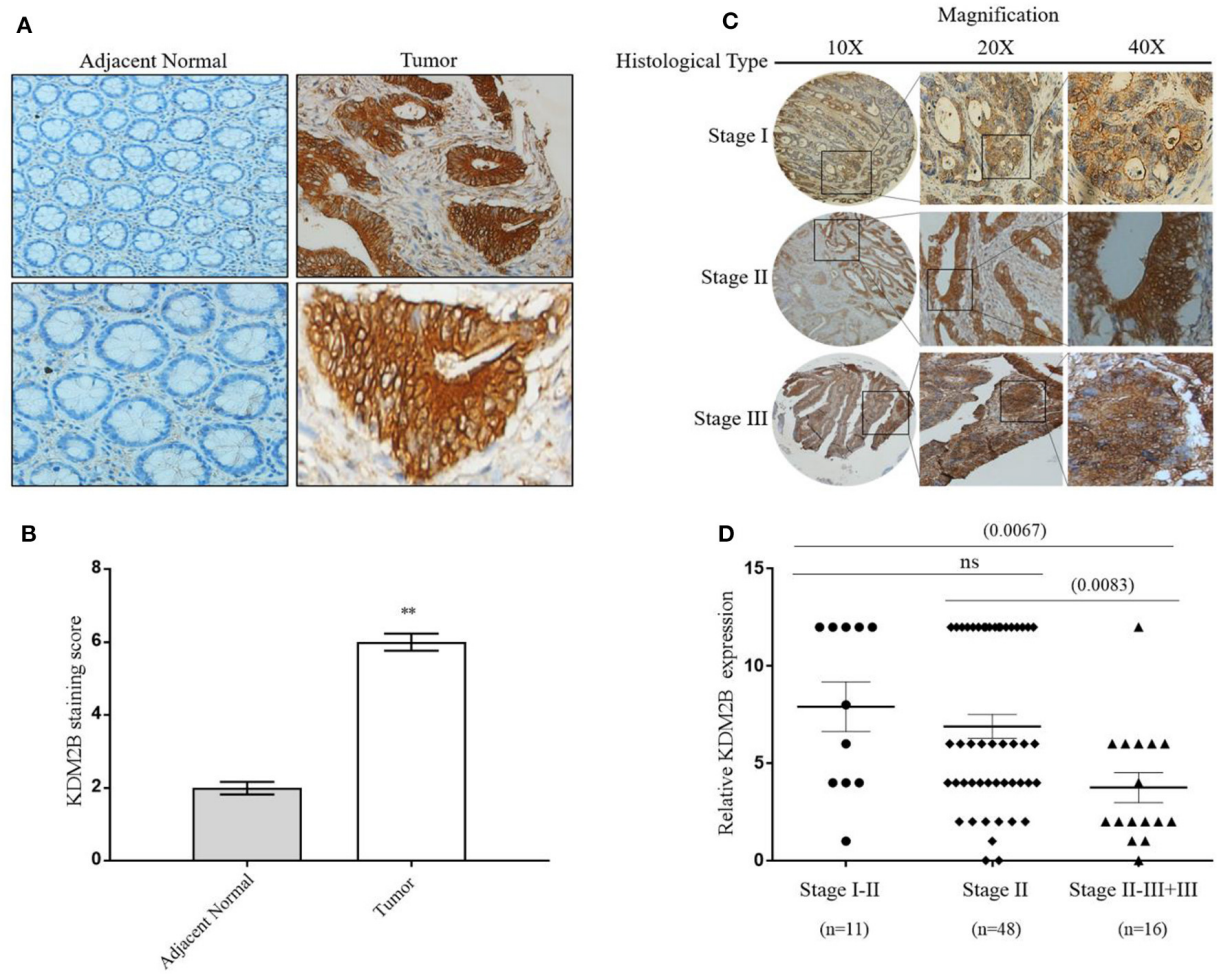
KDM2B is overexpressed in colorectal cancer specimens. **(A)** KDM2B protein was analyzed by immunohistochemistry in the adjacent normal tissue (left) and tumor tissues (right). Cells with brown granules in the nucleus were identified as KDM2B positive. The magnification was × 20 and × 40, respectively. Scale bar = 100 μm. **(B)** The results of KDM2B expression were evaluated by the staining scores. **p < 0.01. **(C)** The images represent differential KDM2B staining intensities in different clinical stages. **(D)** The correlation between the expression of KDM2B and the clinical stage was analyzed between stage I–II, stage II, and stage II–III + III. Stage II–III and Stage III cases were combined into one group. Statistical significant differences were observed between groups (**p < 0.001).

We determined the clinical significance of KDM2B in the development and progression of CRC by analyzing KDM2B in specimens with CRC. We first measured the expression of KDM2B in CRC by IHC analysis using TMA that included 75 tumor tissues (grades I–IV) and 75 normal tissues. Immunohistochemical staining showed that the expression of the KDM2B protein was mainly located at the nucleus of CRC cells (Figure 1A), and its expression was significantly higher in tumor tissues compared with their adjacent normal tissues (*p* < 0.05, Figure 1B). Meanwhile, the positive percentage of KDM2B showed a similar trend in this cohort (*p* < 0.05, Table 1). Furthermore, to investigate the correlation between the expression of KDM2B and the clinicopathological features of CRC, the 75 patient samples were subdivided into two groups [KDM2B (+) positive group and KDM2B (–) negative group] based on the cutoff number of IHC density scores. The chi-square test revealed that the increased expression of KDM2B was strongly related to the clinical stage (*p* = 0.012), and TNM staging classification with statistical significance in T and N (*p* = 0.033, *p* = 0.029, respectively), with no significance in M (*p* = 0.111). No correlation was observed between the expression of KDM2B and sex (*p* = 0.239), age (*p* = 0.525), and tumor size (*p* = 0.380), (*p* > 0.05, Table 2). Also, the correlation between the expression of KDM2B and the CRC clinical stages was analyzed. To examine the correlation between the expression levels of KDM2B and CRC clinical stages, stages II–III and stage III cases were grouped for analysis due to the limited sample numbers. The results showed that the expression levels of KDM2B in stages I–II and stage II are higher than stages II–III + III (*p* < 0.05, Figures 1C,D). These results indicate that KDM2B may serve as a prognostic indicator for patients with CRC.

**Table 1:** Expression of KDM2B protein in the normal colon tissue and colon cancer tissue.

	**No. of cases**	**Positive (%)**	**Negative (%)**	***P-*value**
Normal	75	28 (37.3%)	47 (62.7%)	*P < 0.05
Tumor	75	68 (90.7%)	7 (9.3%)	

Normal group vs. tumor group (*P < 0.05).

**Table 2:** Relationship between KDM2B expression and clinicopathologic parameters in colorectal cancer.

**Characteristics**	**No. of cases**	**KDM2B**		***P*-value**	***X^**2**^***
		**(+)**	**(**−**)**		
**Sex**				0.239	1.384
Female	31	17	14		
Male	44	30	14		
**Age (year)**				0.525	0.405
>55	53	31	21		
≤ 55	22	11	7		
**Tumor size (cm)**				0.380	0.769
>5	46	31	15		
≤ 5	29	16	13		
**Stage**				0.012^*^	12.939
I–II	11	8	3		
II	48	35	13		
II–III	10	3	7		
III	6	1	5		
**T classification**				0.033^*^	4.564
T1+T2	13	11	2		
T3+T4	62	36	26		
**N classification**				0.029^*^	4.748
N0	39	29	10		
N1+N2	36	18	18		
**M classification**				0.111	2.534
M0	65	43	22		
M1	10	4	6		

* *P < 0.05*.

### Silencing KDM2B Inhibited CRC Cell Proliferation and Induced DNA Damage

**Figure 2:**
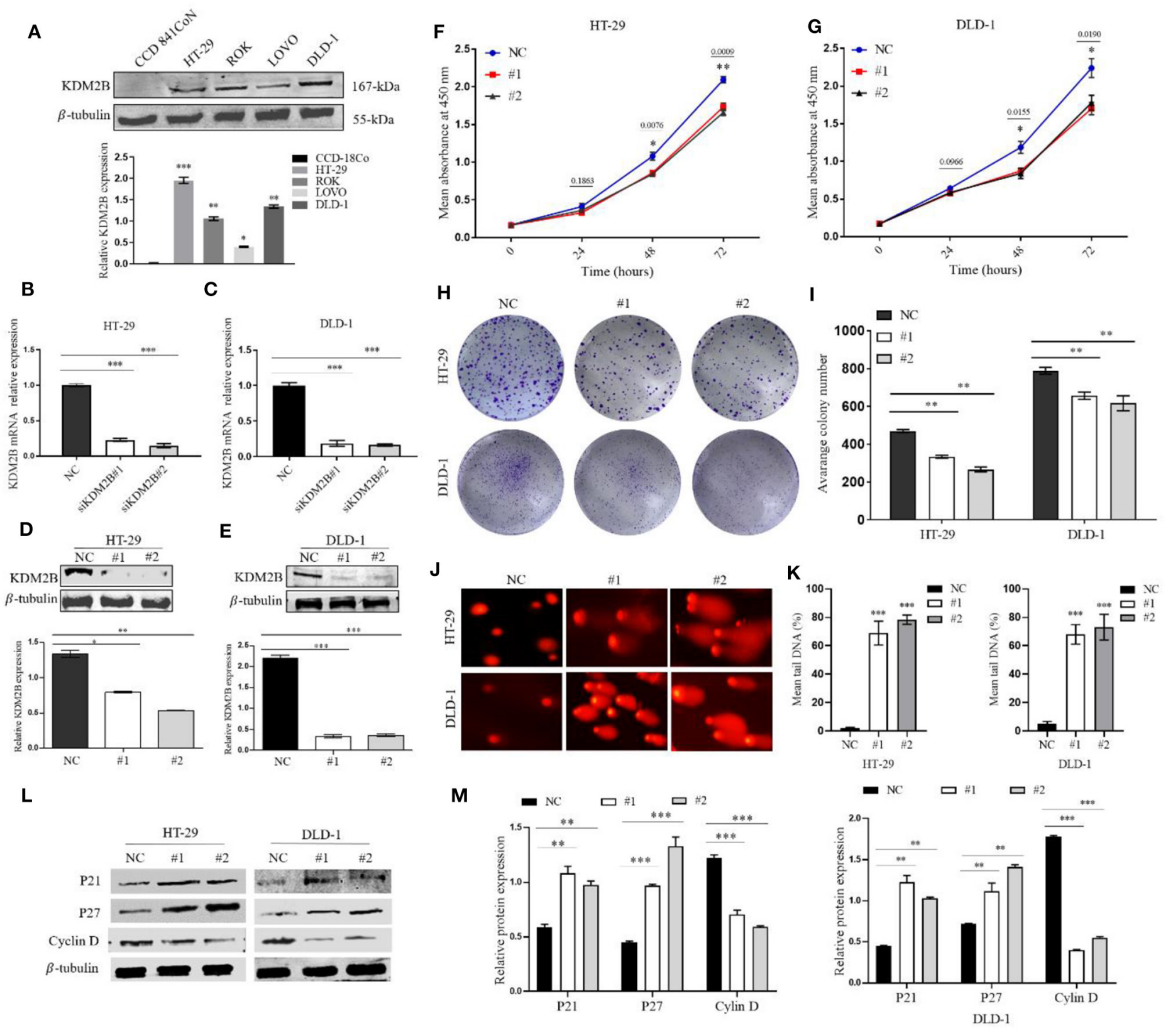
KDM2B restrains cell proliferation in CRC and induces DNA damage. **(A)** KDM2B protein was analyzed by Western blotting in the indicated normal human colon epithelial cells (CCD841CoN) and CRC cell lines (HT-29, ROK, LOVO, and DLD-1) *p < 0.05, **p < 0.01, ***p < 0.001. **(B,C)** HT-29 and DLD-1 were transfected with siRNA (siKDM2B #1 and siKDM2B #2) and negative control (NC) using lipofectamine 2000. The relative expression of KDM2B mRNA was examined by real-time qRT-PCR after normalizing to GAPDH (*n* = 3), ***p < 0.001. **(D,E)** The assessment of the expression level of KDM2B protein and the bar chart of quantified KDM2B protein expression in transfected HT-29 and DLD-1 cells. Data are presented as Mean ± SEM (*n* = 3). Statistical significant differences in mRNA and protein in cells were observed (*p < 0.05, **p < 0.01, ***p < 0.001 *vs*. negative control). The results of the CCK-8 assay **(F,G)** demonstrated that cell viability decreased in HT-29 and DLD-1 cells after KDM2B knockdown *p < 0.05, **p < 0.01 *vs*. control. **(H,I)** HT-29 and DLD-1 cell colonies formed and graphical presentation of the average of colonies formed in control (NC) and transfected groups (#1 and #2). Data are expressed as Mean ± SEM (*n* = 3). **p < 0.01. **(J)** Comet images from HT-29 and DLD-1 cells after knockdown of KDM2B. The transfected cell group shows increasing levels of damage compared with the negative control. The number of cells scored in each measured concentration was 50. **(K)** The bar chart of the mean tail comet in percentage *** *p* < 0.001. **(L)** Representative densities of P21, P27, Cyclin D, and β-Tubulin proteins after Western blot experiment. **(M)** Cluster bar charts of representative proteins **p < 0.01, ***P < 0.001.

To assess the impact of the expression of KDM2B on the biologic features and tumorigenesis of CRC cells, KDM2B protein expression was first analyzed in five different cell lines, normal human colon epithelial cell lines (CCD841CoN), and human CRC cell lines (HT-29, ROK, LOVO, DLD-1). The expression of KDM2B was highly expressed in human cancer cell lines compared with the normal human colon epithelial cell line (Figure 2A). Then, HT29 and DLD1 cells were chosen to knockdown KDM2B expression by siRNA. The qRT-PCR and Western blotting were performed to validate the efficiency of the knockdown. The expression levels of KDM2B mRNA significantly decreased with siRNA transfection (siRNA#1 and siRNA#2) compared to levels in the control group (Figures 2B,C). Moreover, Western blotting showed that KDM2B protein expression was also significantly reduced with siRNA#1 and siRNA#2 treatment compared to the control group (Figures 2D,E).

Given that KDM2B delays cancer cell growth by inducing senescence and/or apoptosis, we sought to determine whether KDM2B could be inducing DNA damage in CRC cells, resulting in delayed cell growth and altering the regulation of its downstream proteins that are key to the survival and growth of tumor cells. We first examined the cell proliferative capacity by CCK-8 and colony formation. The CCK-8 assay and colony formation assay showed that the downregulation of KDM2B significantly retarded the growth and colony formation of HT-29 and DLD-1 cells compared to the negative control (Figures 2F–I).

To determine the possible reason for the inhibition of KDM2B inducing cell proliferation depilation, DNA damage was conducted using the comet assay. The results illustrated in Figures 2J,K showed increased DNA tail in the transient KDM2B knockdown group compared to the negative control, concluding that the knockdown of KDM2B induces DNA damage in CRC cells. Next, to confirm the regulatory mechanism of KDM2B on the survival and proliferation of CRC cells, cell proliferative related proteins P21, P27, and cyclin D1 were examined by Western blot assay. The result shows that the downregulation of KDM2B significantly increased protein levels of P21; P27, meanwhile, decreased the protein level of cyclin D1 (Figures 2L,M). Collectively, these observations suggest that the knockdown of KDM2B might inhibit proliferation in CRC cells in response to DNA damage and activation of proliferative-related proteins P21, P27, and cyclin D.

### The Downregulation of KDM2B in CRC Cells Is Associated With Stem Cell Features

**Figure 3:**
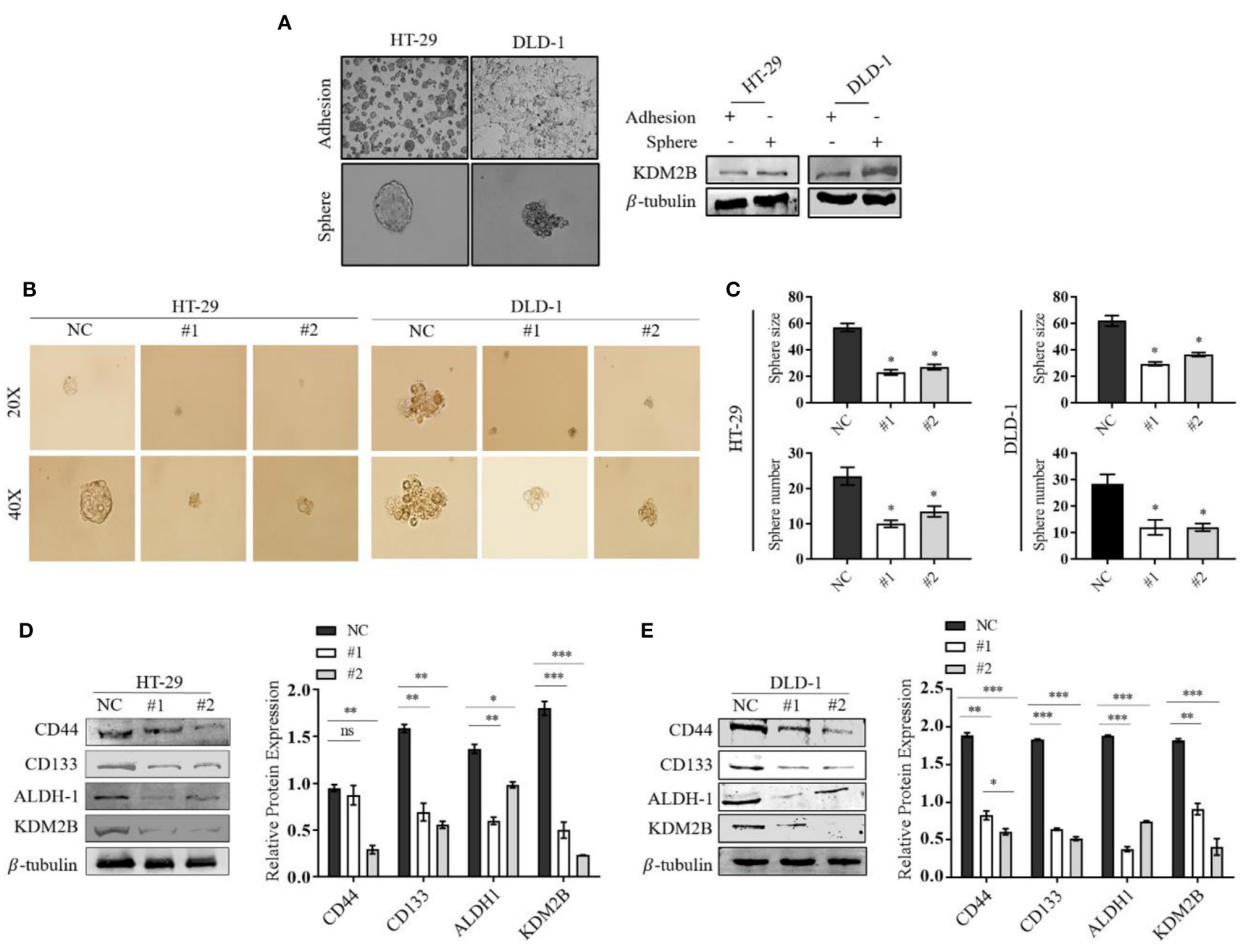
The downregulation of KDM2B regulates the cell stemness in CRC in CRC cell lines. Representative images of adherent cells and the tumor sphere. **(A)** The assessment of the expression level of KDM2B protein in adherent cells and the tumor sphere was analyzed by Western blotting. **(B)** Tumor sphere formation of HT-29 and DLD-1 cells. Cells were treated with siKDM2B (#1 and #2) or negative control (NC) for 7 days, and expansion of the tumor spheres were analyzed at 20 × and 40 × magnification under a microscope (bar = 50 μm; magnification, 200 × and 400 ×). **(C)** The cluster bar chart of the number and size of spheres formed. **(D,E)** The effect of KDM2B on CRC cell stem-like markers. CD44, CD133, ALDH-1, and KDM2B levels were analyzed by Western blotting. Data are expressed as Mean ± SEM (*n* = 3). *p < 0.05, **p < 0.01, ***p < 0.001 *vs*. control.

The role of KDM2B in CRC stem cells remains unclear. To determine whether the downregulation of KDM2B in CRC is associated with stem-like properties, the sphere formation assay was performed and measured the differential expression of the KDM2B protein. The Western blot results showed that the protein level of KDM2B was significantly higher in spherical cells compared to their counterpart adherent cells (Figure 3A). We then downregulated the expression of KDM2B in HT-29 and DLD-1cells using siKDM2B. Our results showed decreased tumor-sphere size and the number of spheres in both CRC cell lines following siRNA transfection compared with the negative control (Figure 3B). The quantification of these findings is shown in Figure 3C.

The surface markers CD133, CD44, and ALDH-1 have been proposed for the identification and characterization of CRC-CSCs. Western blot results showed that the downregulation of KDM2B correlated with decreased expression levels of CD133, CD44, and ALDH-1 in transfected HT-29 and DLD-1 cells compared with the negative control (Figures 3D,E). These results suggest that the knockdown of KDM2B may inhibit cell stemness in CRC.

### The Downregulation of KDM2B Decreased the Expression of EZH2 and Regulated the Activity of the PI3K/AKT Signaling Pathway

**Figure 4:**
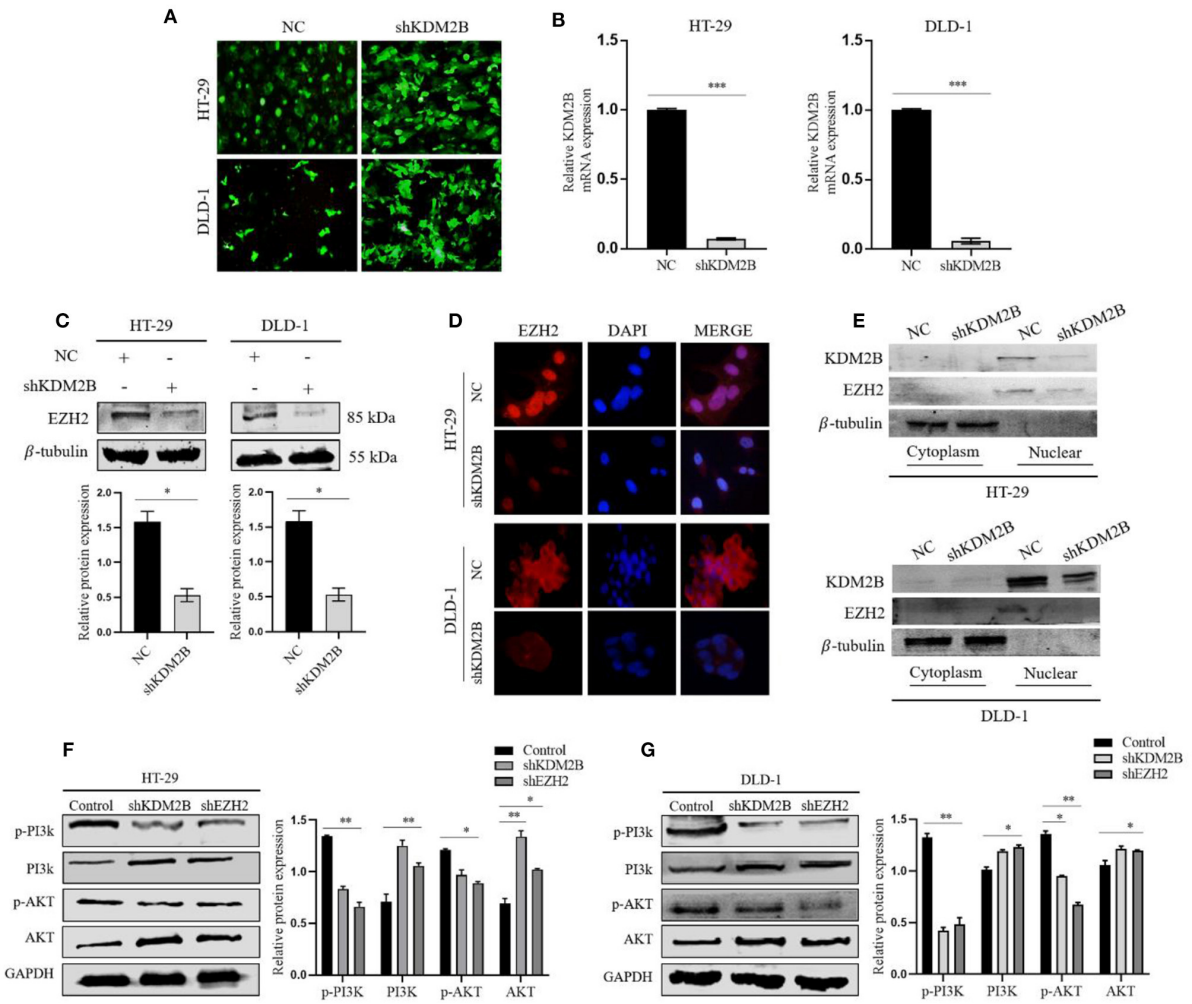
KDM2B suppresses the expression of EZH2 in CRC and activates the PI3K/AKT signaling pathway. **(A)** HT-29 and DLD-1 cells were infected with shRNA against KDM2B (shKDM2B) and negative control (NC), and more than 90% of cells had green fluorescence under the fluorescence microscope. Representative microscopic pictures were taken. **(B)** The relative expression of KDM2B mRNA was examined by real-time qRT-PCR after normalizing to GAPDH (*n* = 3), ***p < 0.001. The expression of EZH2 protein was determined by Western blotting and immunofluorescence after transfecting KDM2B as described in **(A,C,D)**. **(C)** Shows Western blot result and representative quantification of EZH2 expression in HT-29 and DLD-1. β-tubulin was used as a loading control. **(D)** Immunofluorescence was performed using FITC-labeled phalloidin. The cells were stained with anti-KDM2B and anti-EZH2 antibodies (red) and DAPI (blue) and observed under a fluorescence microscope (Scale bar: 20 μm, magnification, 40 ×). **(E)** The cytosolic and nuclear proteins were extracted from HT-29 and DLD-1 transfected cells with shKDM2B or control vectors (NC) and the protein levels of KDM2B and EZH2 were measured using Western blot analysis. β-tubulin was used as an internal control. **(F,G)** The downregulation of KDM2B and EZH2 activated the PI3K/AKT signaling pathway. The expression levels of PI3K/AKT pathway target genes, including p-PI3K, PI3K, p-AKT, and AKT, were analyzed by Western blot Assay. GAPDH was used as a loading control. The data was statistically significant at *p < 0.05, **p < 0.01, ***p < 0.001 as compared to negative control. The data correspond to the mean ± SEM of three independent experiments.

To elucidate the functional relationship between KDM2B and EZH2 in CRC, we selectively silenced the KDM2B gene *via* the lentiviral expression vector of short hairpin RNA (shRNA) against KDM2B (shKDM2B) and negative control (NC) in HT-29 and DLD-1 cells. The inhibition efficiency of KDM2B was verified by a fluorescence microscope and real-time qRT-PCR analyses. We observed that more than 90% of cells had green fluorescence under the fluorescence microscope (Figure 4A). KDM2B mRNA expression in shKDM2B-HT-29 and shKDM2B-DLD-1 groups reduced compared to the negative control (Figure 4B). Moreover, the expression of EZH2 was subsequently detected by Western blot analysis and immunofluorescence. Figures 4C,D show that the expression of EZH2 was markedly reduced by inhibition of KDM2B (shKDM2B) at protein levels compared with negative control (NC).

To further probe the regulating roles of KDM2B on the transcription of EZH2, we analyzed the impact of KDM2B on the cytoplasm and nuclear protein levels of EZH2. Western blot results showed that the knockdown of KDM2B decreased the protein levels of EZH2 in the cytoplasm and increased the levels in the nucleus (Figure 4E). These data indicate that KDM2B regulates the expression of EZH2 in CRC cells.

Activation of the PI3K-AKT pathway is crucial in cancer progression. To investigate whether the KDM2B and EZH2 could regulate the PI3K/Akt signaling pathway, we examined the effect of KDM2B and EZH2 on downstream proteins of the PI3K/Akt pathway. As shown in Figures 4F,G, downregulating KDM2B and EZH2 in HT-29 and DLD-1 cells decreased the expression of phosphorylated PI3K and phosphorylated AKT and increased the expression of PI3K and AKT, suggesting that the downregulation of KDM2B and EZH2 activates the PI3K-AKT signaling pathway in CRC.

### KDM2B and EZH2 Were Indispensable for the Maintenance of Stem Cells in CRC *in vitro via* Regulating the PI3K/AKT Pathway

**Figure 5:**
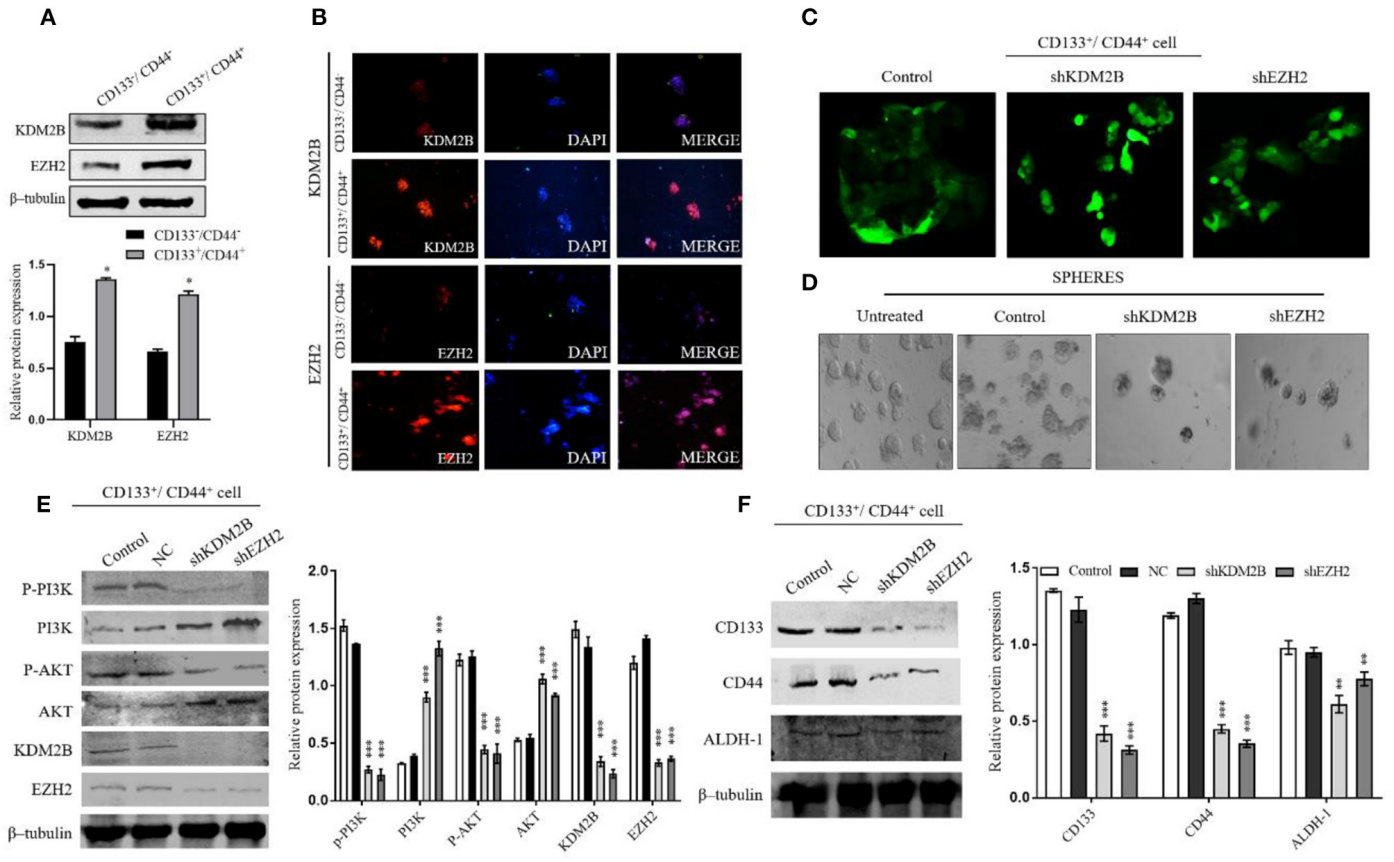
KDM2B and EZH2 were indispensable for CCS-like cell maintenance *in vitro via* regulating the PI3K/AKT pathway. The CRC cancer stem cell population (CD133^−^/CD144^−^ and CD133^+^/CD144^+^) were isolated from HT-29 cells by MACS. **(A)** Protein expression levels of KDM2B and EZH2 confirmed in CD133^−^/CD144^−^ and CD133^+^/CD144^+^ cells by Western blot. The cluster bar chart represents the Western blot quantification **(A)** *p < 0.05. **(B)** Immunofluorescence staining of KDM2B and EZH2 in CD133^−^/CD144^−^ and CD133^+^/CD144^+^ cells (Scale bar: 100 μm). **(C)** CD133^+^/CD144^+^ cells were transfected *via* the lentiviral expression vector of short hairpin RNA (shRNA) against KDM2B and EZH2 (shKDM2B and shEZH2) and negative control, and more than 70% of cells had green fluorescence under the fluorescence microscope. Representative microscopic images were taken. **(D)** Sphere formation in untreated, control, shKDM2B, and shEZH2 groups. The expansion of tumor spheres was analyzed at 40 × magnification under a microscope (bar = 50 μm; magnification, 400 ×). The knockdown of KDM2B and EZH2 activated the PI3K/AKT pathway in the stem cell population in CRC. **(E)** The expression of p-PI3K, PI3K, p-AKT, AKT, KDM2B, and EZH2 was analyzed by Western blot Assay after KDM2B and EZH2 knockdown in CD133^+^/CD44^+^ cell population. β-tubulin was used as a loading control. **(F)** The downregulation of KDM2B and EZH2 weakens self-renewal markers in CRC cells. The assessment of protein expression of stemness markers including CD44, CD133, and ALDH-1 was analyzed by Western blotting. The data was statistically significant at **p < 0.01, ***p < 0.001 as compared to the control. Data are represented as mean ± SEM of three independent experiments.

KDM2B and EZH2 play an important role in the maintenance of the self-renewal capacity and tumorigenic ability of CSCs. We hypothesized that the relationship between KDM2B and EZH2 might influence CRC-CS–like cells. Thus, to accredit this hypothesis, we sorted the CD133^+^/CD44^+^ and CD133^−^/CD44^−^ subpopulations from HT-29 cells by magnetic-activated cell sorting and analyzed the expression of KDM2B and EZH2. The protein levels of KDM2B and EZH2 were higher in the CD133^+^/CD44^+^ cells than in CD133^−^/CD44^−^ cells (Figures 5A,B). The expression of KDM2B and EZH2 were detected by Western blot analysis and immunofluorescence (Figures 5A,B). We then transfected CD133^+^/CD44^+^cells with NC, shKDM2B, and shEZH2 and performed a sphere formation assay to test the self-renewal capacity of the cells, which is a pivotal property of CS-like cells *in vitro*. The results demonstrated that more than 70% of cells had green fluorescence under the fluorescence microscope (Figure 5C), and the sphere colonies were fewer in shKDM2B and shEZH2 groups compared with the control group (CD133^+^/CD44^+^cells without transfection) and negative control (NC) (Figure 5D).

Given that EZH2 and KDM2B are essential in the maintenance of CSC and that both participate in the activation of the PI3K-AKT signaling pathway, we sought to determine whether the PI3K-AKT pathway is involved in the EZH2 and KDM2B-mediated regulation of the cell stemness of CRC. We analyzed the downstream proteins of the PI3K-AKT signaling pathway, finding that the CD133^+^/CD44^+^ population showed increased AKT and PI3K expression after knocking down KDM2B and EZH2 (Figure 5E). Moreover, surface markers CD133, CD44, and ALDH-1 showed decreased expression in shKDM2B and shEZH2 groups as compared with the control group (untreated cell) and negative control (NC) (Figure 5F). These results suggest that KDM2B and EZH2 regulate the cell stemness in CRC *via* the PI3K-AKT pathway.

### KDM2B and EZH2 Inhibit Migration and Invasion in CRC Cells

**Figure 6:**
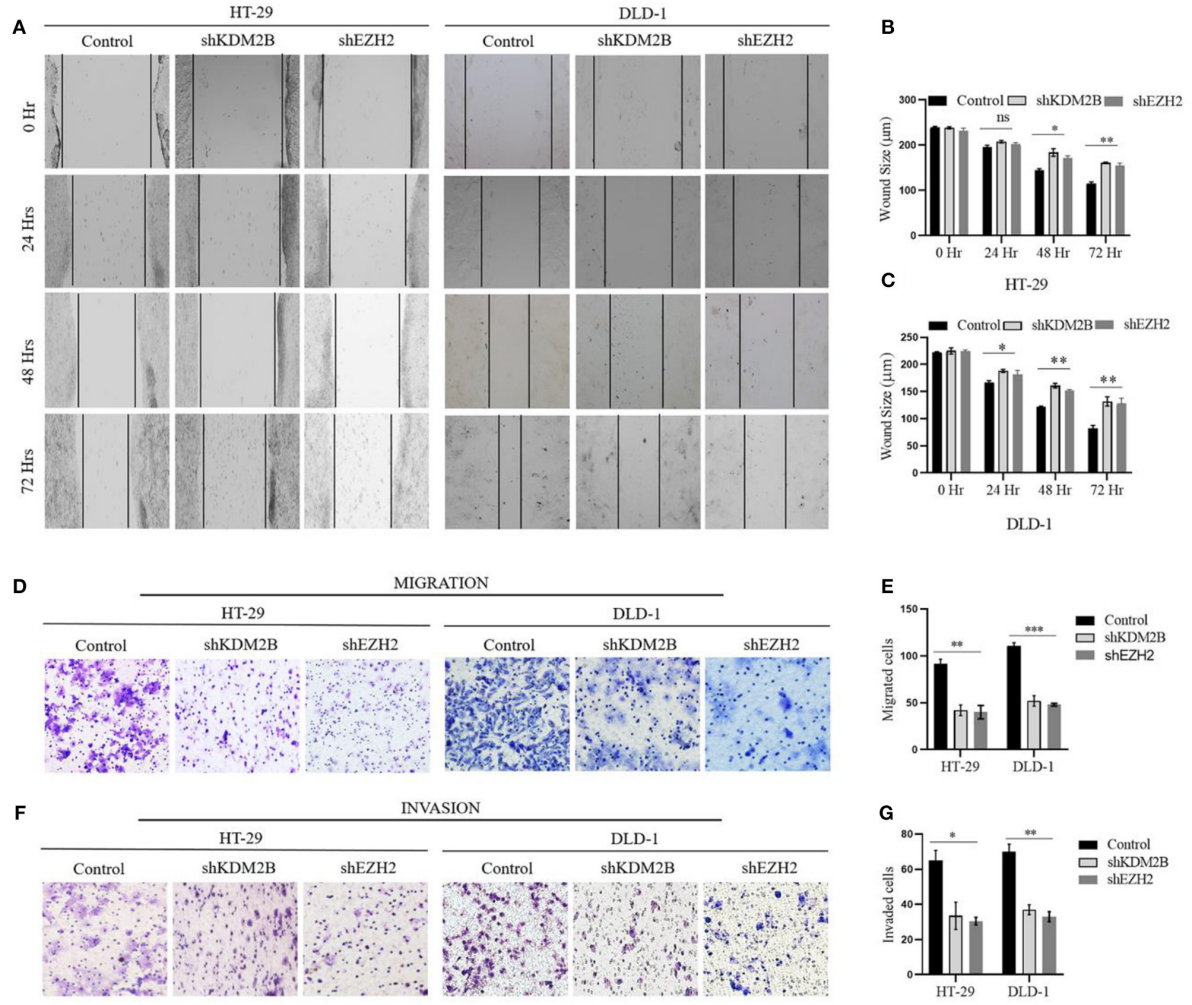
The downregulation of KDM2B and EZH2 inhibits the migration and invasion in CRC cells. HT-29 and DLD-1 cells were treated with either shKDM2B or shEZH2. **(A–G)** The migration and invasion abilities of the cells were examined by scratch wound healing assay, migration, and invasion assay (Transwell assay). **(A)** A representative image of the wound area after the scratched wound healing assay. **(B,C)** The cluster bar chart of the wound area in μm at various time points. **(D,F–H)** Representative pictures and the cluster bar chart of migrated and invaded HT-29 and DLD-1 cells. The data was statistically significant at *p < 0.05, **p < 0.01, ***p < 0.001 as compared to control. Data are represented as mean ± SEM of three independent experiments.

Next, we aimed to interrogate whether the downregulation of KDM2B and EZH2 influences cell migratory and invasive capacities of CRC using scratch wound healing and Corning transwell assays. Scratch wound healing experiment determined the extent of cell migration by inferring to close or narrowing of the wound area in culturing cells at time points of 24, 48, and 72 h in HT-29 and DLD-1 cells, respectively. Our results showed wound area closure was lower in shKDM2B and shEZH2 groups compared to the control group, following 48 and 72 h of incubation (Figure 6A). The quantification of these findings is shown in Figures 6B,C. Subsequently, we assessed the impacts of KDM2B and EZH2 on the cell migration and invasion of CRC using Corning transwell assays. Also, migrated and invaded cells were remarkably inhibited by KDM2B and EZH2 knockdown as shown in Figures 6D,E. The differences in migration observed in the control and cells treated with shRNA were significant (Figures 6F,G). These results indicate that KDM2B and EZH2 inhibit cell migratory and invasive capacities in CRC.

### Correlation of KDM2B With EZH2 in Tissues With CRC

**Figure 7:**
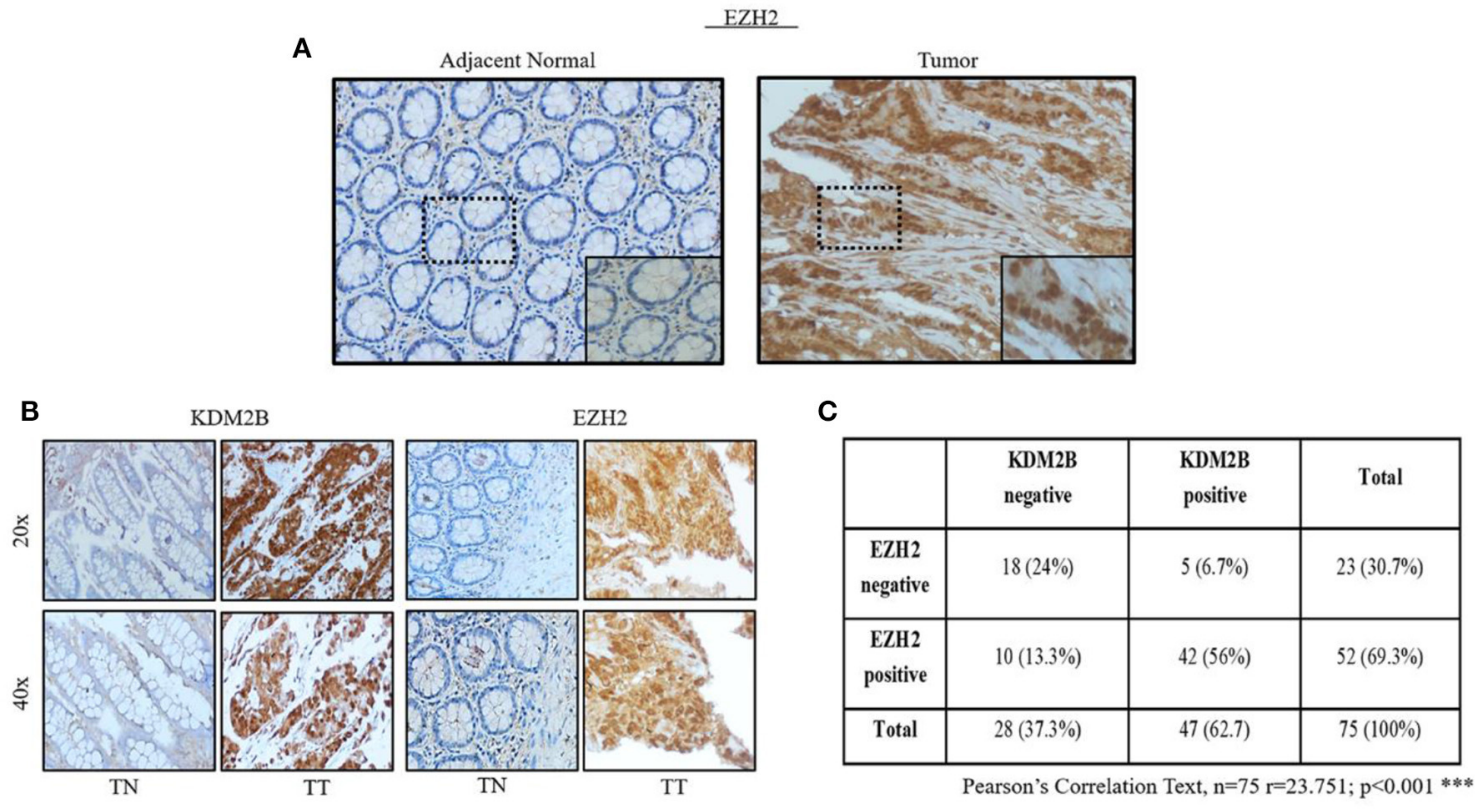
The expressions of EZH2 is positively related to KDM2B in tissues with CRC. **(A)** Representative images of the expression of EZH2 in adjacent normal tissue specimens with CRC *vs*. tumor tissue. **(B)** The expression of KDM2B and EZH2B proteins in TMA tissue sections was represented by the cohort. The magnification was × 20 and × 40, respectively. Scale bar = 100 μm. **(C)** The negative correlation of KDM2B with the expression of EZH2 was assessed using the chi-square Pearson correlation text (*n* = 75; *r* = 23,751; ***p < 0.001).

Our study demonstrated that KDM2B transcriptionally decreased the expression of EZH2 in CRC cells, and both seem to play an important role in the maintenance of the self-renewal of CR-CSCs. To further confirm our results, we analyzed the protein levels of EZH2 in TMA of CRC. The same cohorts of TMA sections for KDM2B were immunostained with a specific anti-EZH2 antibody. The result showed that EZH2 was highly expressed in the nucleus of the tumor tissue compared with adjacent normal tissue (Figure 7A). We then analyzed the correlated expression of EZH2 and KDM2B in human tissues with CRC. The result demonstrated a positive correlation between the expression of EZH2 and KDM2B in human tissues with CRC (Figures 7B,C) (Pearson correlation test: *r* = 23.751, *p* < 0.001).

## Discussion

Cancer stem cells have recently been shown to present a serious approach for establishing therapeutic strategies targeting CSCs (7). Accumulating studies have shown that CR-CSCs can initiate tumorigenesis and recurrence of CRC (38). Hence, a better understanding of the tumor environment and strategies for the eradication of CR-CSCs is needed. In this study, we investigated the functional role of KDM2B in CRC and the influence of KDM2B and EHZ2 on the characteristic of CR-CSCs regarding the self-renewal ability of CRC cells *via* the PI3K/AKT signaling pathway.

KDM2B has been considered as either a tumor suppressor or ontogenesis depending on the cellular context (17, 18). To determine the expression pattern of KDM2B in CRC, we first investigated the expression of KDM2B in specimens with CRC. We found that the expression of KDM2B increased in tumor tissues compared with normal tissues. Consistent with previous studies, KDM2B has been reported to be overexpressed in gastric cancer and glioma and its expression correlated with cancer progression (39, 40). Additionally, we analyzed the correlation between the expression of KDM2B and the pathological features of CRC. The overexpression of KDM2B highly correlated with the CRC clinical-stage grade and TNM staging. The correlation between the expression of the relative KDM2B and clinicopathological staging was also analyzed. From stage I to stage III in CRC, the expression of KDM2B was higher in tumor tissues than that of the corresponding adjacent-normal tissues in each stage, and this difference exhibited an important significance in stages I–II and stage II than stage II–III + III. Our result showed that the average expression of KDM2B in tumor tissues that are in stages I–II and stage II was higher than that of stages II–III + III. These results indicate that KDM2B may affect the early process during the initiation and progression of CRC and might play a vital role in the development of CRC.

KDM2B contrib-utes to uncontrolled cell growth by inhibiting tumor suppressor genes and promotes the expression of oncogene (18, 19). A previous study reported that the downregulation of KDM2B inhibits cell proliferation and affects the progression of the cell cycle in tumor cells including HeLa cells (17). Moreover, KDM2B regulates gene transcription *via* the demethylation of H3K36me2 (14). The methylation of H3K36 is involved in several nuclear processes such as transcriptional regulation, gene dosage compensation, DNA replication, recombination, and DNA damage repair (41, 42). Staberg et al. (24) demonstrated that the loss of KDM2B induces DNA damage and apoptosis and sensitizes glioblastoma cells to chemotherapy. In accordance with those studies, we determined the functional role of KDM2B in CRC. Our data showed that the downregulation of KDM2B inhibited cell growth in CRC, induced DNA damage, and decreased proliferative proteins p21, p27 while increasing the expression of cyclin D1. This may lead to the conclusion that KDM2B may function as an oncogene in CRC, and KDM2B plays a vital role in tumorigenesis and progression.

In addition to regulating cell proliferation, for the first time, our present study showed that the knockdown of KDM2B also regulates the stemness in CRC. Defined markers were utilized for the identification of CR-CSCs. Researchers have demonstrated that CD133, CD44, ALDH1, CD166, and Lgr5 are critical biomarkers to identify CR-CSCs, where the combination of CD133 with CD44 seems to be more reliable as target biomarkers (5–7). Our data showed that the knockdown of KDM2B was significantly associated with the properties of CSCs, such as self-renewal. We found KDM2B to be expressed at higher levels in spherical cells compared to adherent cells. Furthermore, the knockdown of KDM2B suppressed sphere formation and inhibited CR-CSC markers including CD133, CD44, and ALDH-1 in CRC cells. Consistent with a previous study, KDM2B was highly expressed in glioblastoma stem cells (GSCs) compared to their differentiated counterparts. Moreover, the enrichment of GSCs through the cell surface marker, CD133, revealed preferential expression of KDM2B in GSCs compared to non-GSCs (24). Another study reported NDY1/KDM2B functions as a master regulator of Polycomb complexes and controls the self-renewal of breast cancer stem cells (13). These findings indicate that KDM2B modulates cell survival and the self-renewal of CSCs.

There is mounting evidence that highlights the role of KDM2B in the regulation of stem cell transcription factor, EZH2 (23, 32). EZH2 has tumor-suppressive functions affecting tumor cell proliferation, invasion, or metastasis (25–27). To link KDM2B to stem cell regulatory pathways, we queried stem cell regulatory pathways that have been linked to the maintenance of stem cells in CRC. Thus, we tested the effect of KDM2B on the expression of EZH2 in CRC cells. Our data demonstrated that the downregulation of KDM2B reduced the transcriptional activity of the expression of EZH2 in the CRC cell line. Recently, Zacharopoulou et al. (43) reported reduced expression of EZH2 and BMI1 in HCT-116 by KDM2B. This finding reveals KDM2B as a key regulator of the expression of EZH2. In addition, KDM2B seems to regulate the EZH2 levels through several pathways (32). The PI3K/AKT signaling cascade is one of the most important intracellular pathways, which regulates survival, cell growth, differentiation, and cellular metabolism (33, 34). EZH2 and PRC2-mediated H3K27me3 (trimethylation at lysine 27 of histone H3) contribute to transcriptional silencing, and this process is negatively regulated by PI3K/AKT (44). Previous data demonstrated a mechanism by which PI3K/AKT signaling modulates the cancer epigenome through controlling H3K4 methylation in breast cancer (33). However, the mechanism by which PI3K/AKT signaling network regulates the expression of KDM2B remains unclear. Emphasizing the interest in this particular signaling pathway, we examined the effect of KDM2B and EZH2 on the downstream proteins of the PI3K/Akt pathway in CRC cells. Our data showed that the downregulation of KDM2B and EZH2 activated PI3K/Akt signaling.

A previous study reported the role of EZH2 in CRC-CS–like cell properties by activating Wnt/βcatenin signaling (31). Given that the downregulation of KDM2B and EZH2 influences the PI3K/Akt pathway activation might be linked to the regulation of CR-CSCs, we analyzed their influences in the properties of CR-CSCs. The protein expressions of KDM2B and EZH2 were detected in CD133^+^/CD44^+^ cells and CD133^−^/CD44^−^ cell populations. Interestingly, the protein expressions of KDM2B and EZH2 were highly expressed in CD133^+^/CD44^+^ cells as compared to CD133^−^/CD44^−^ cells. In addition, the knockdown of KDM2B and EZH2 reduced sphere formation in CD133^+^/CD44^+^ cells, concluding that both KDM2B and EZH2 influence CRC stem-like cells. The PI3K/Akt pathway plays an important role in the cell sphere formation and growth of colon cancer stem (35). Here, we demonstrated a critical mechanism for the activation of the PI3K/Akt pathway by KDM2B and EZH2 in CR-CSCs. The expression of the PI3K/Akt downstream proteins PI3K and AKT was also increased in CD133^+^/CD44^+^ cells after knocking down KDM2B and EZH2 expressions. Additionally, the downregulation of KDM2B and EZH2 increased the surface markers CD133, CD44, and ALDH-1. Collectively, our data showed that the regulation of KDM2B and EZH2 of the cell stemness in CRC is likely *via* the activation of the PI3K-AKT pathway.

Furthermore, we determined the functional role of KDM2B and EZH2 in the cell migration and invasion in CRC. We found that the knockdown of KDM2B and EZH2 suppressed the cell migration and invasion in CRC. In line with our result, Kottakis et al. (45) reported that FGF-2 regulates cell proliferation, migration, and angiogenesis through an NDY1/KDM2B-miR-101-EZH2 pathway.

Our study found that KDM2B transcriptionally regulates EZH2 in CRC cells, and both seem to play an important role in the maintenance of the self-renewal of CR-CSCs. To further confirm our results, we analyzed the expression of EZH2 and KDM2B in the CRC array with the same cohort, and the Pearson correlation test showed a positive correlation between EZH2 and KDM2B. This finding highlights a novel mechanism in which KDM2B transcriptionally decreased the expression of EZH2, and both seem to play important role in CRC and the features of CRC stem cells.

## Conclusion

In summary, our research found that KDM2B is highly expressed in CRC and is correlated with the clinical stage and TNM staging. The downregulation of KDM2B inhibited cell proliferation and induced DNA damage. KDM2B decreased the expression of EZH2 contributing to the activation of the PI3K/AKT signaling pathway. Mechanically, KDM2B and EZH2 could orchestrate the stemness in CRC *via* activating the PI3K/AKT pathway. The knockdown of KDM2B and EZH2 impeded the cell migration in CRC. The expression of EZH2 positively correlated with KDM2B in the malignancy of CRC. This finding suggests that KDM2B and EZH2 play a key role in the stemness of CRC and, therefore, could be a potential therapeutic target for CRC.

## Data Availability Statement

The original contributions presented in the study are included in the article/Supplementary Material, further inquiries can be directed to the corresponding author/s.

## Ethics Statement

The human tissue samples of patients diagnosed with colorectal cancer were used in this research. Patients provided written informed consent, and the experimental protocols were approved by the Committee for the Ethical Review of Research, The Second Affiliated Hospital of Dalian Medical University (No. 2021, 001).

## Author Contributions

JS and LL designed the study and wrote the manuscript. JS and QZ performed the experiments. JS, XC, XL, YZ, JM, YL, and JH analyzed the data. IY, MN, and BS revised the manuscript and assisted in the study design. All the authors read and approved the final manuscript.

## Conflict of Interest

The authors declare that the research was conducted in the absence of any commercial or financial relationships that could be construed as a potential conflict of interest.
