# Supplementary material for: The Role of KDM2B and EZH2 in Regulating the Stemness in Colorectal Cancer Through the PI3K/AKT Pathway
Source: Front Oncol. 2021 Mar 9;11:637298. doi: 10.3389/fonc.2021.637298 (PMC8006351; doi:10.3389/fonc.2021.637298)
Supplement: Supplementary file 1 [file Data_Sheet_1.pdf]

Gel Uncropped

The protein band images were captured with ODYSSEY infrared imaging system (Version 3.0 software, LI-COR Biosciences). The protein quantitation was calculated by Image J software, then the graph pad was used to design the graphs and to evaluate differences between groups.

1. KDM2B expression in normal epithelial cell CCD841CoN and colorectal cancer cells Ht-29, ROK, LOVO and DLD-1 (**Figure 2**).

KDM2B

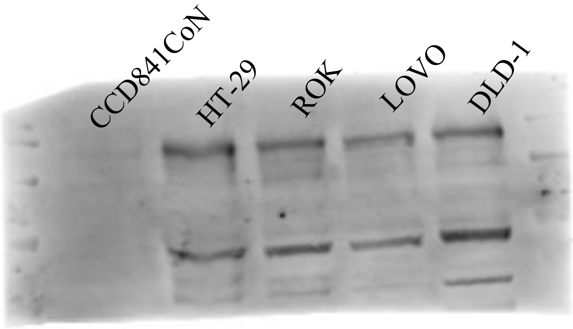

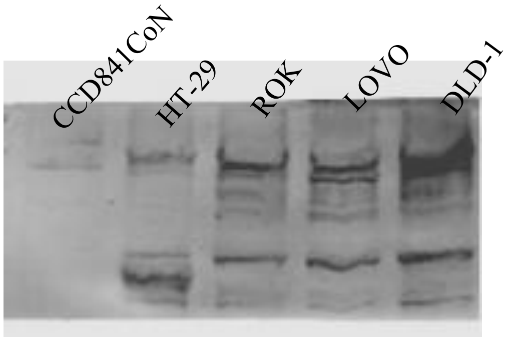

KDM2B

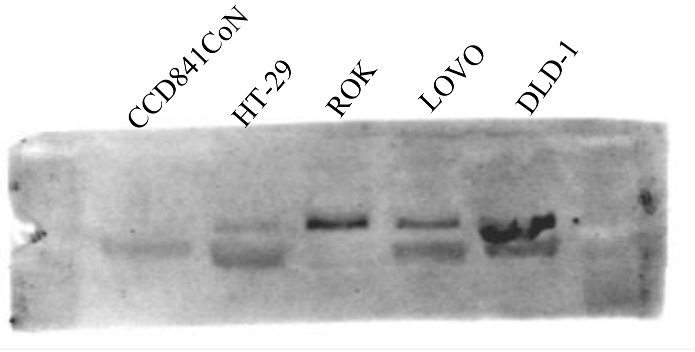

Beta- tubulin

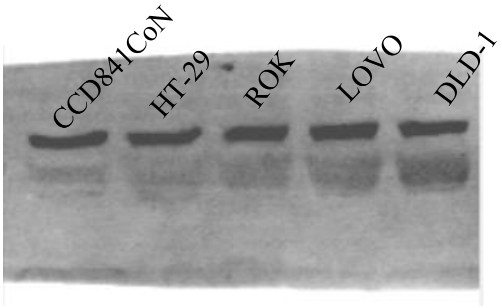

2. Knockdown of KDM2B by siRNA against KDM2B in HT-29 and DLD-1 cells (**Figure 2**).

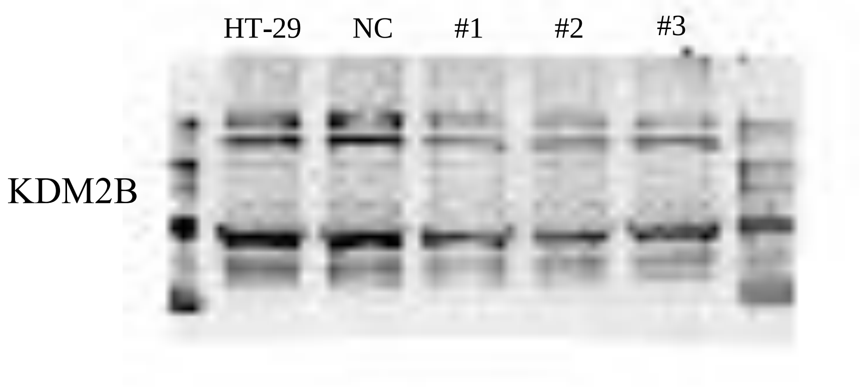

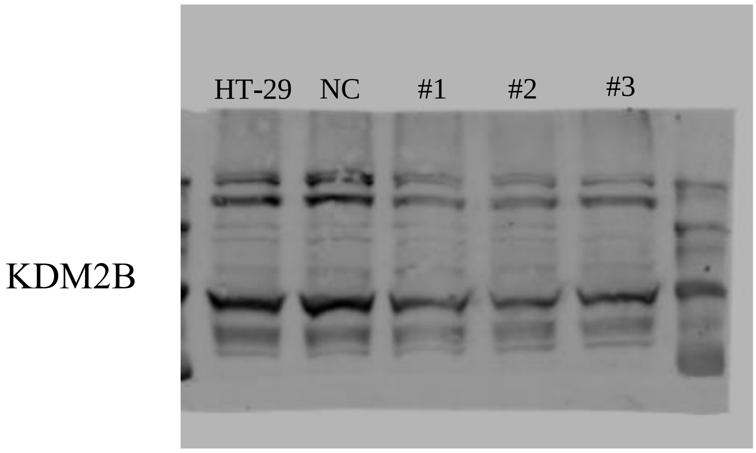

Beta- tubulin

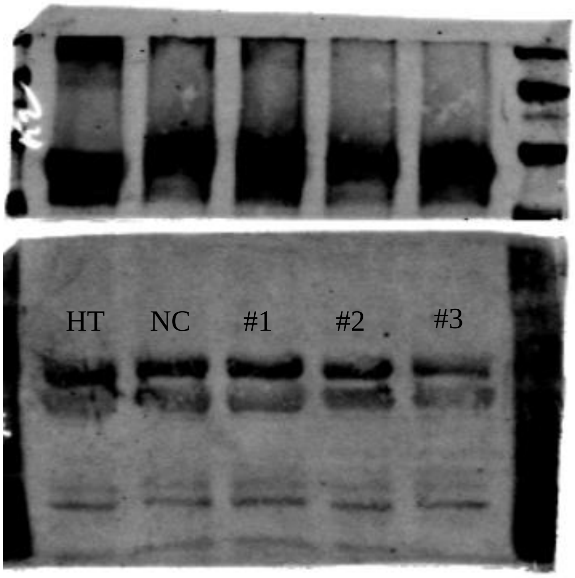

KDM2B

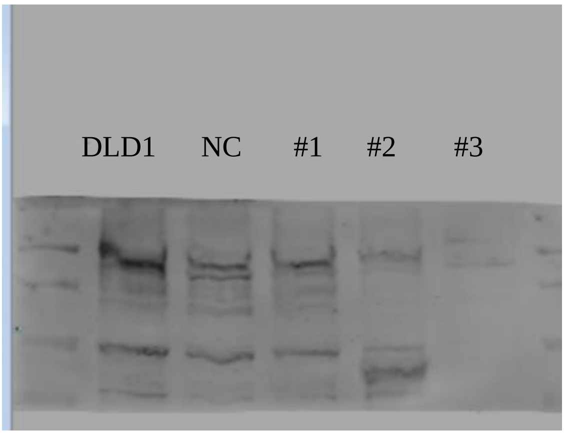

Beta- tubulin

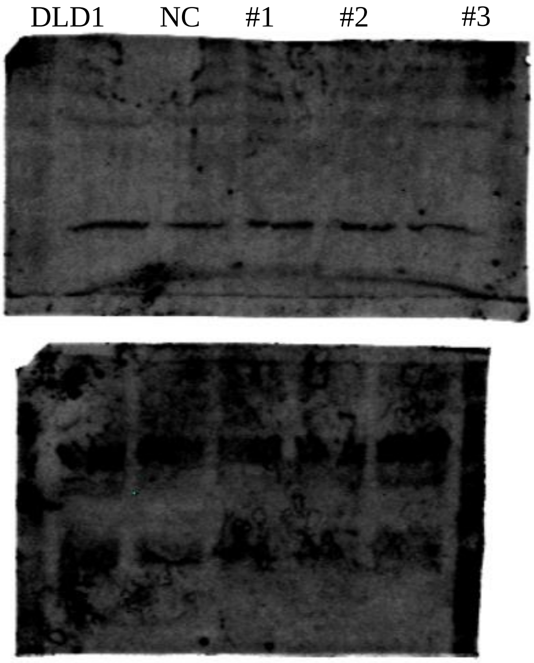

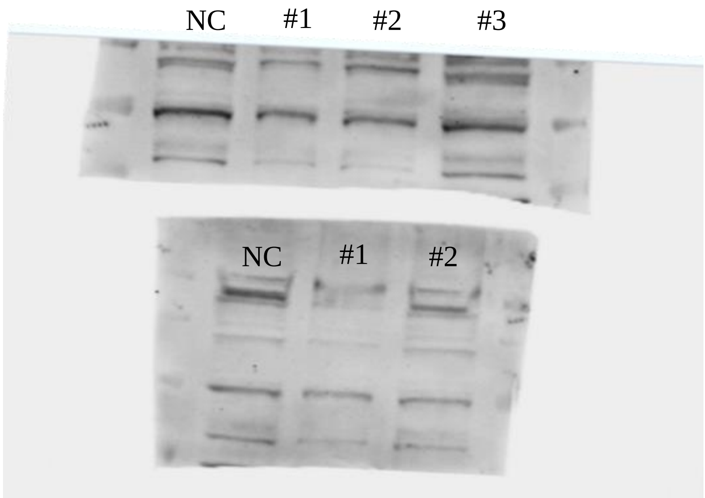

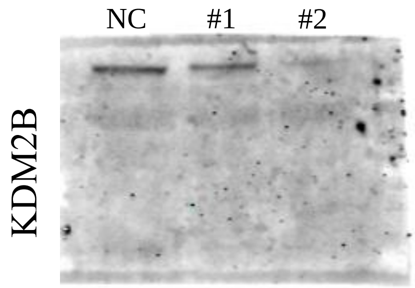
NC
#1
#2
KDM2B

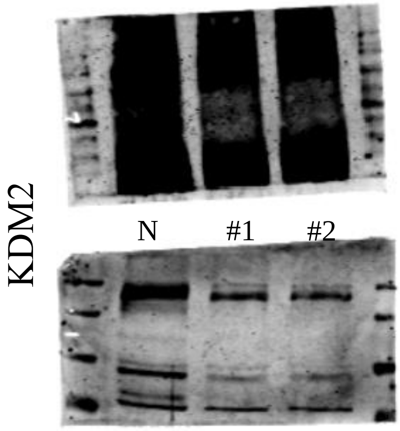
KDM2
N
#1
#2

3. Representative protein expression of P21, P27, Cyclin D, and β-Tubulin proteins in HT-29 and DLD-1. (**Figure 2**)

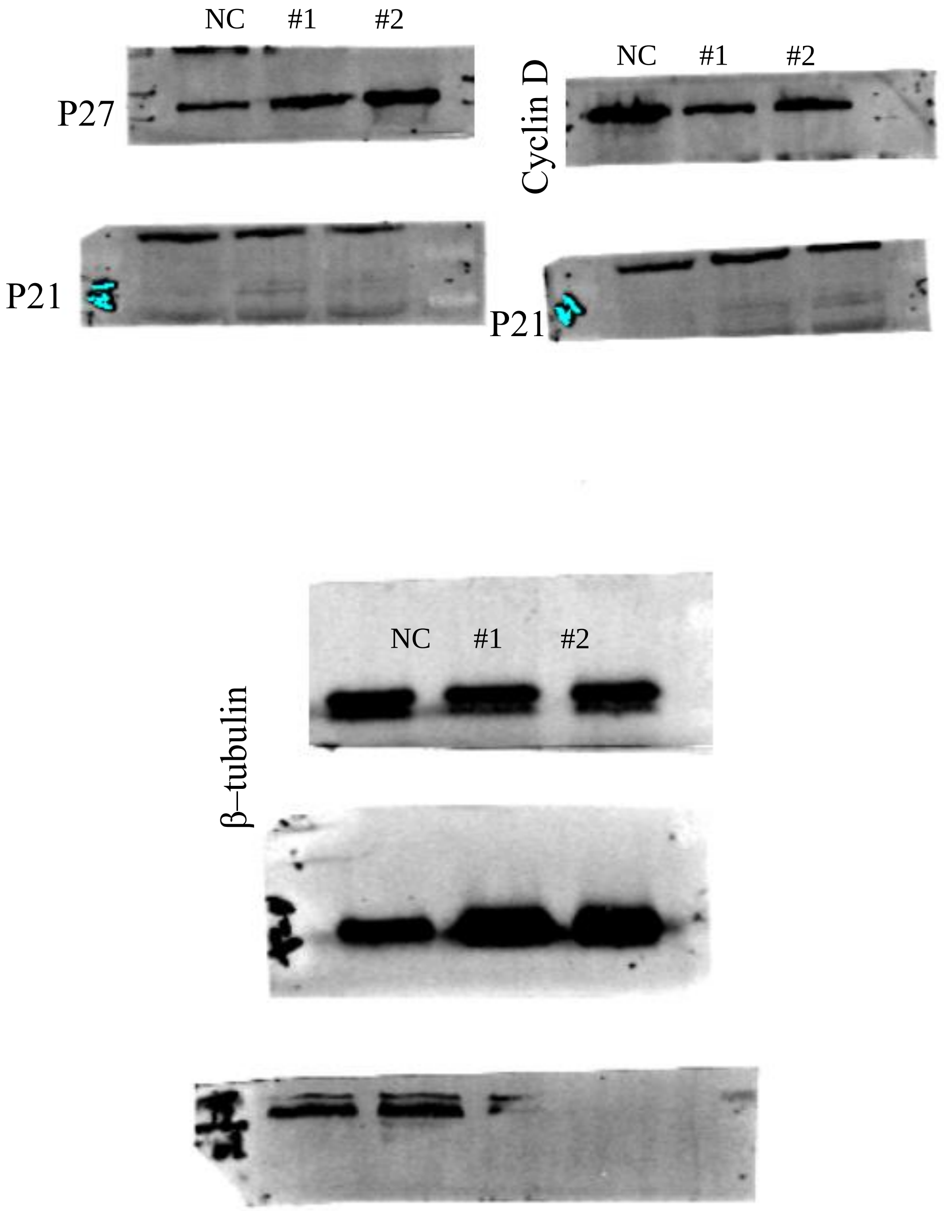

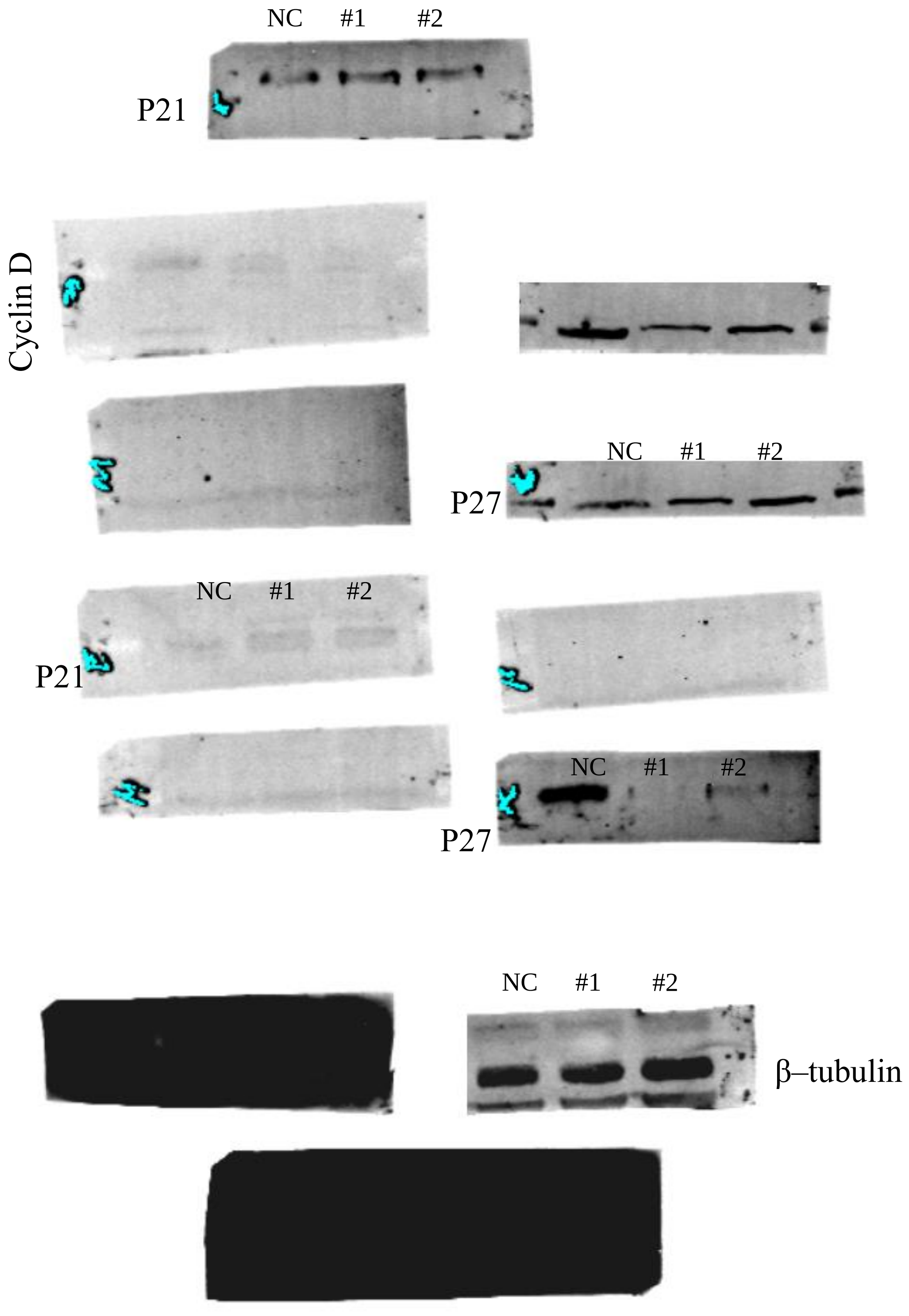
NC #1 #2
P21
Cyclin D
NC #1 #2
P27
NC #1 #2
P21
NC #1 #2
P27
NC #1 #2
β–tubulin

4. Assessment of KDM2B protein expression level in adherent cells and tumorsphere (**Figure 3**)

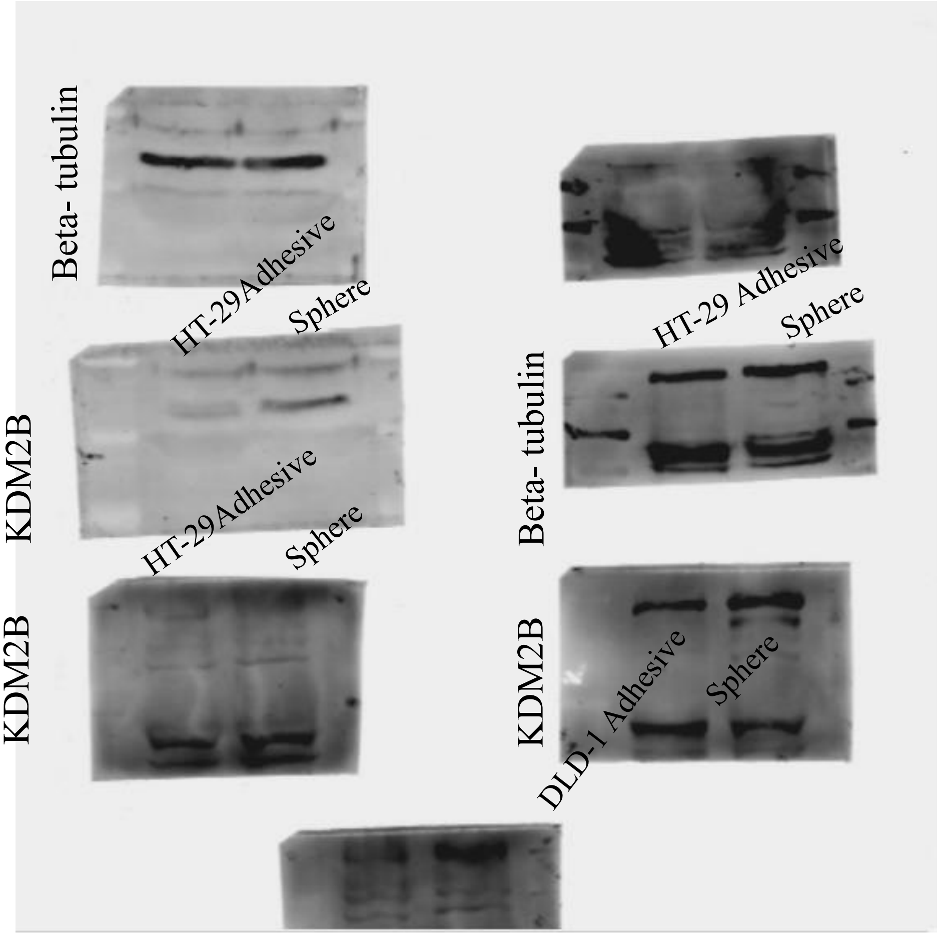

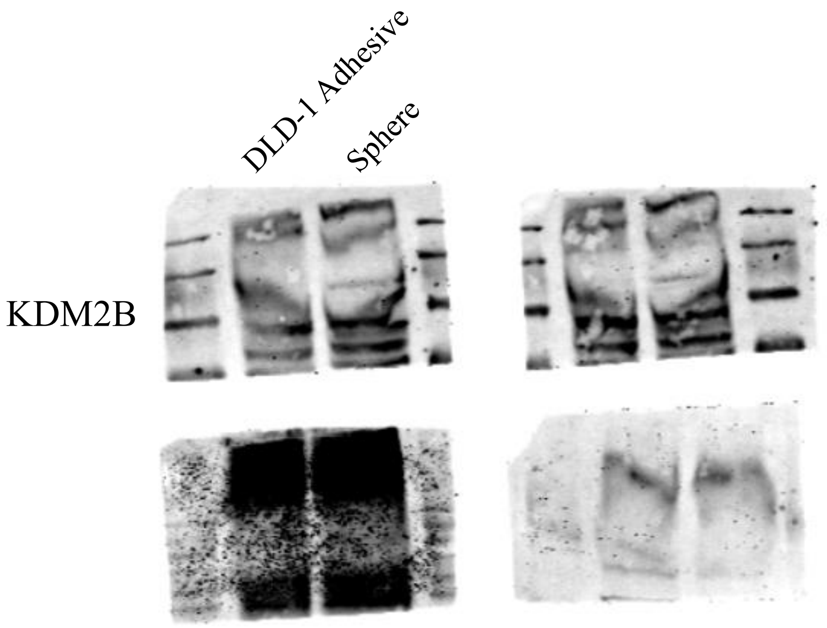
DLD-1 Adhesive
Sphere
KDM2B

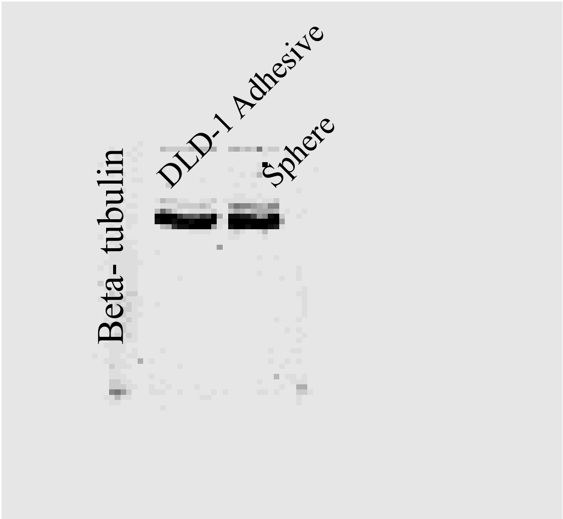
Beta- tubulin
DLD-1 Adhesive
Sphere

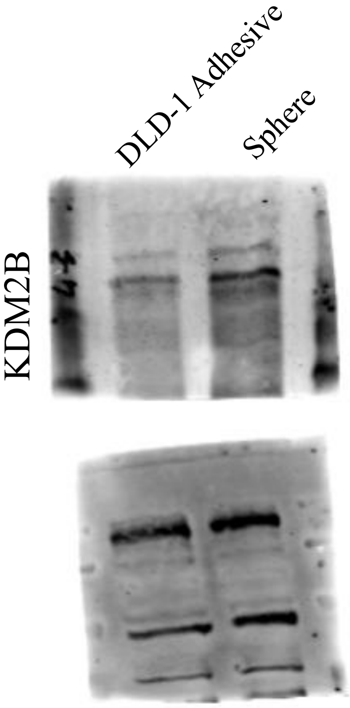
DLD-1 Adhesive
Sphere
KDM2B

5. Expression KDM2B on CRC cell stem-like markers, CD44, CD133, ALDH-1, and KDM2B levels (**Figure 3**)

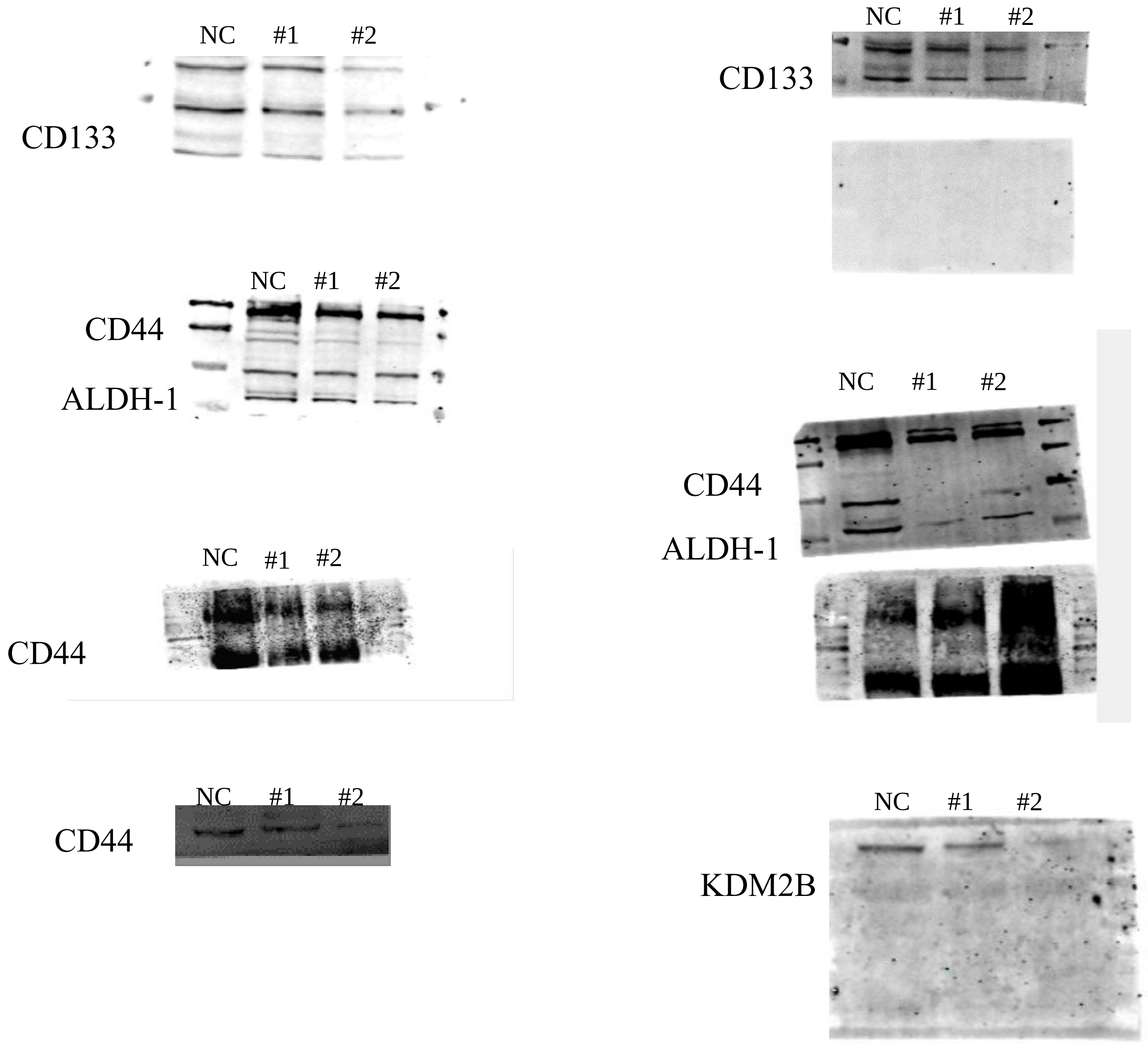

Beta- tubulin

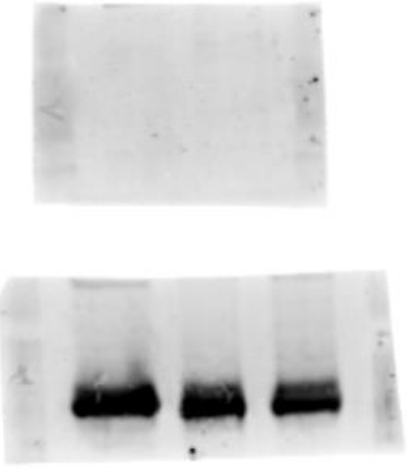

Beta- tubulin

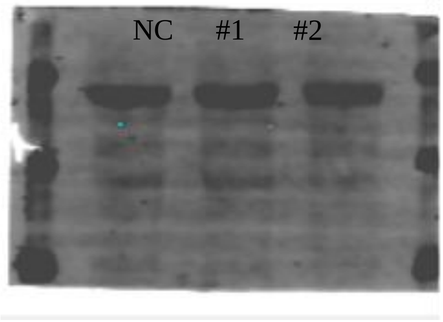

ALDH-1

ALDH-1

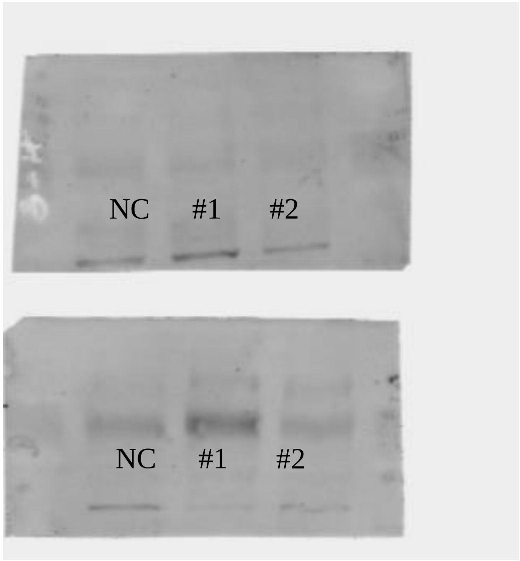

6. Western blot result of EZH2 expression in HT-29 and DLD-1 following knockdown of KDM2B. β-tubulin was used as a loading control (**Figure 4**).

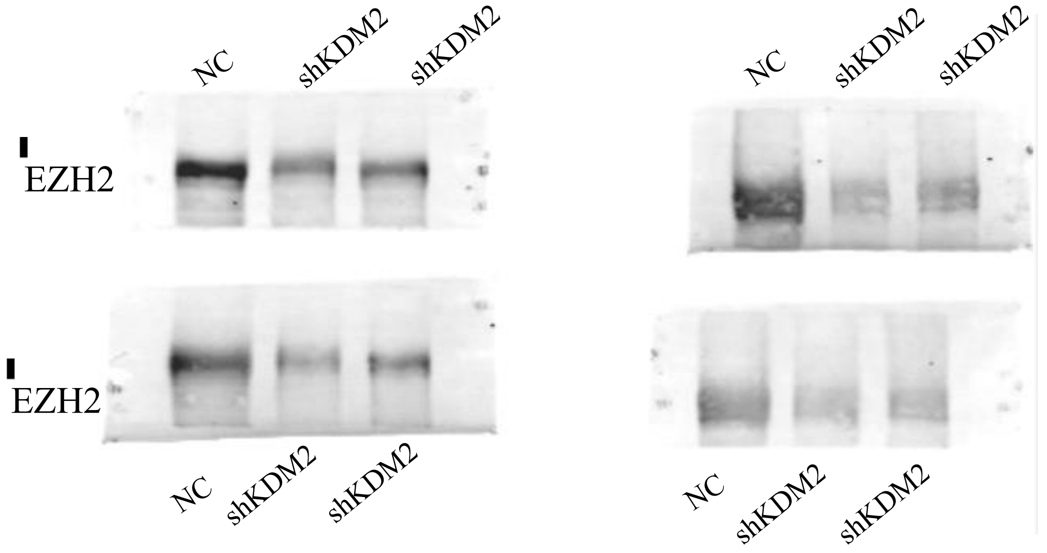

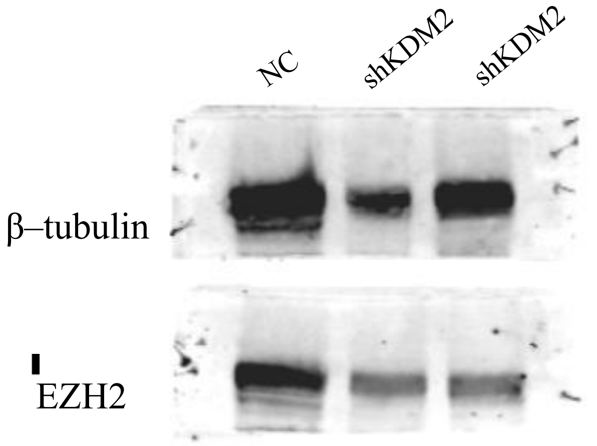

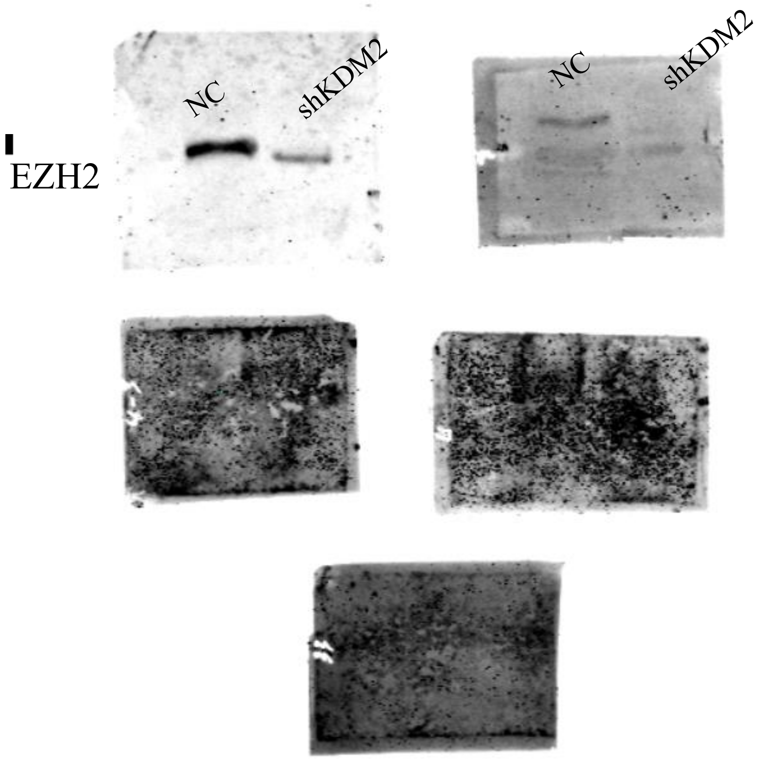
NC
shKDM2
EZH2
NC
shKDM2

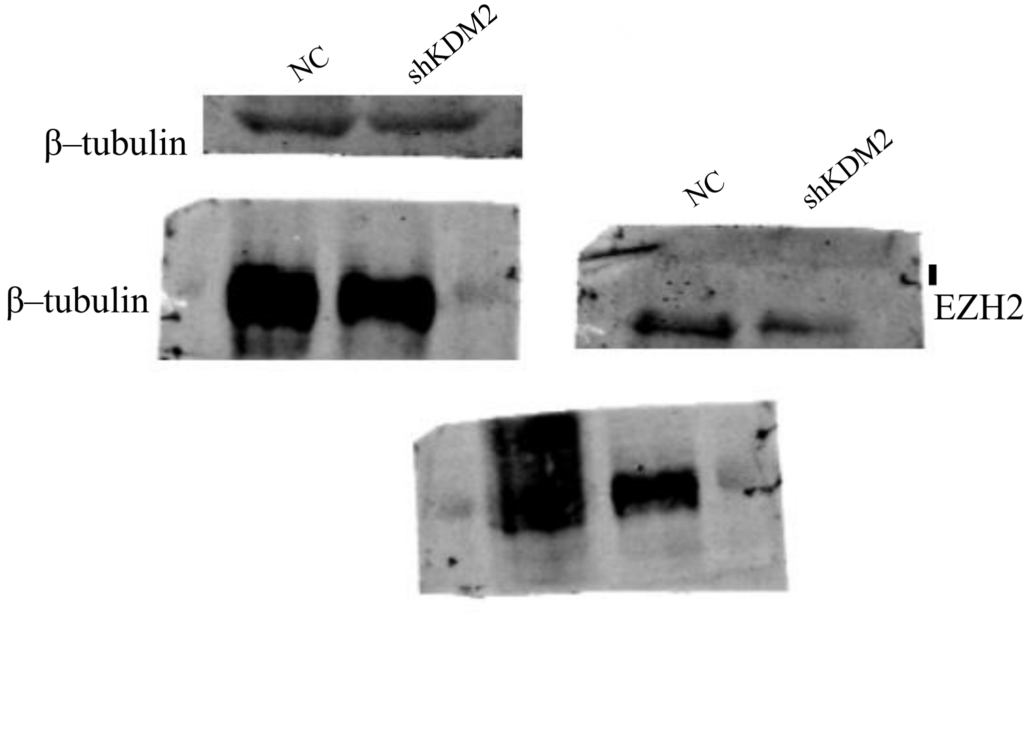
NC
shKDM2
β–tubulin
β–tubulin
NC
shKDM2
EZH2

7. The cytosolic and nuclear proteins expression of KDM2B and EZH2 in HT-29 and DLD-1 transfected cells with shKDM2B or control vectors (NC) (**Figure 4**)

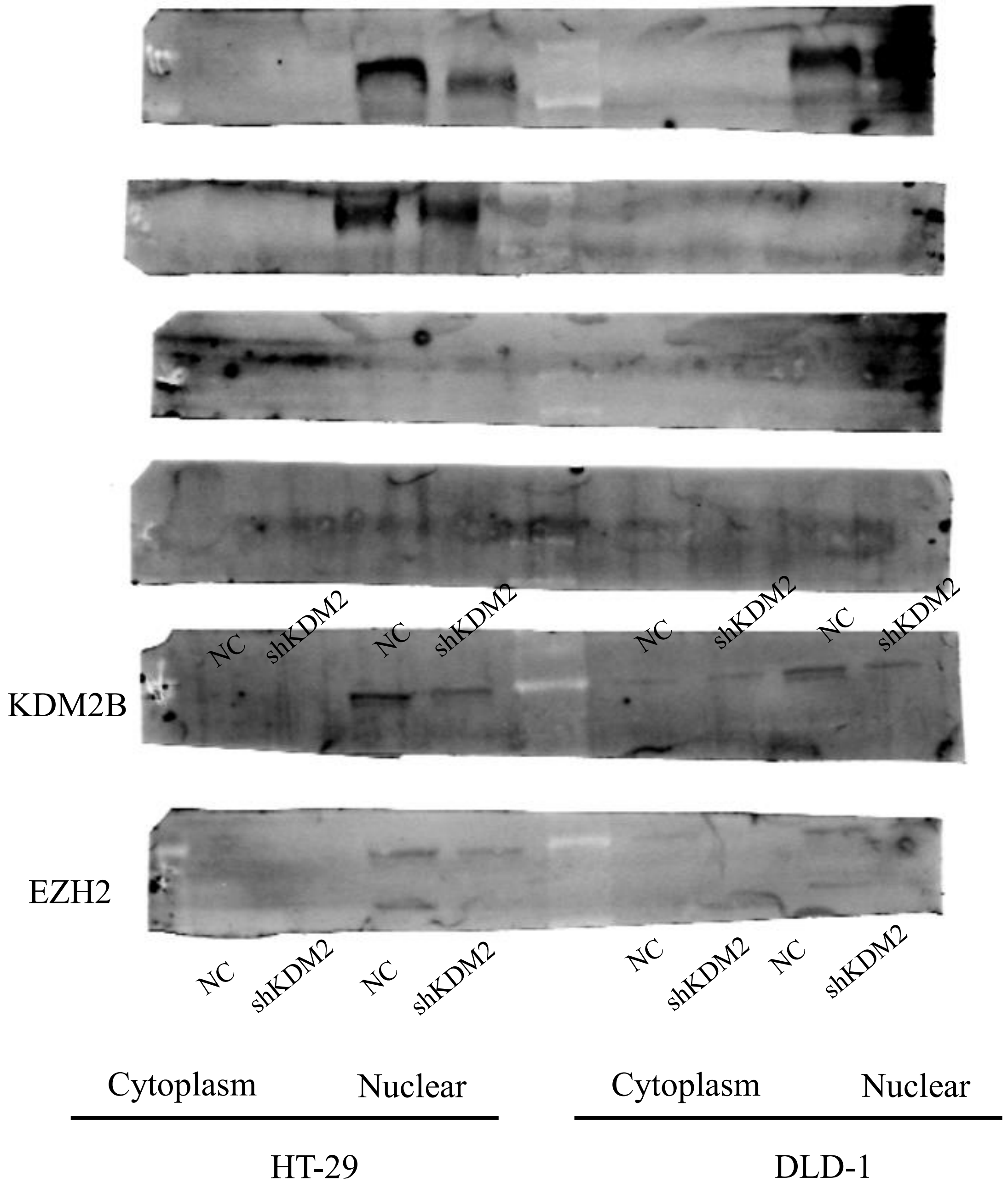

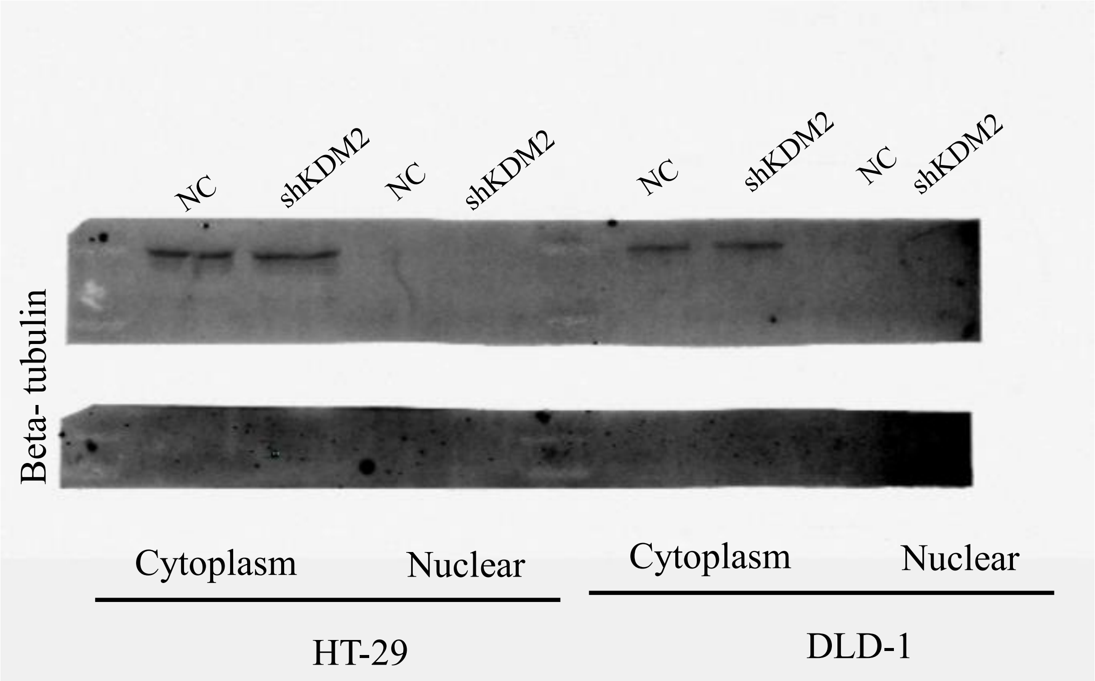
NC
shKDM2
NC
shKDM2
NC
shKDM2
NC
shKDM2
Beta- tubulin
Cytoplasm
Nuclear
Cytoplasm
Nuclear
HT-29
DLD-1

8. The expression levels of PI3K/AKT pathway target genes in HT-29 and DLD-1, including p-PI3K, PI3K, p-AKT, AKT. GAPDH was used as a loading control (**Figure 4**)

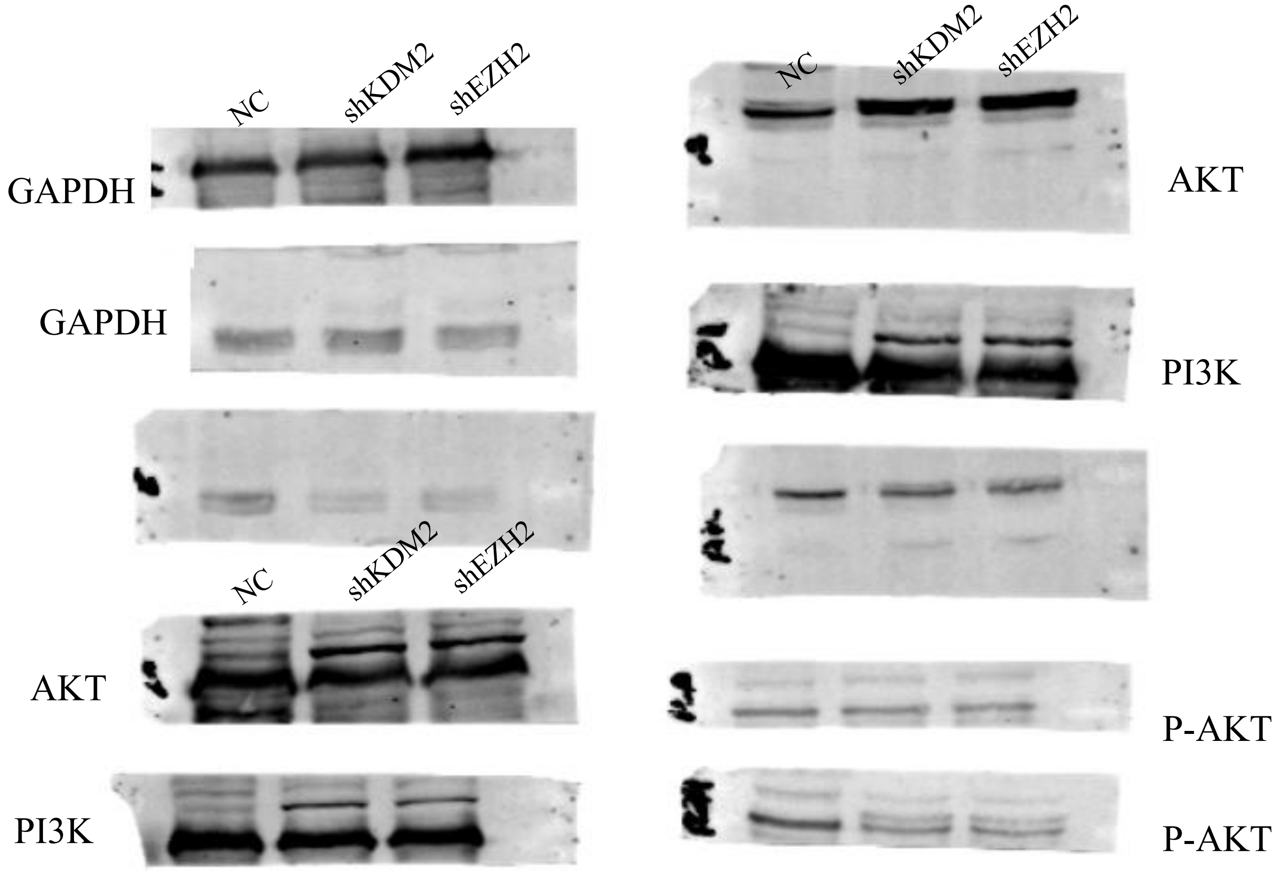

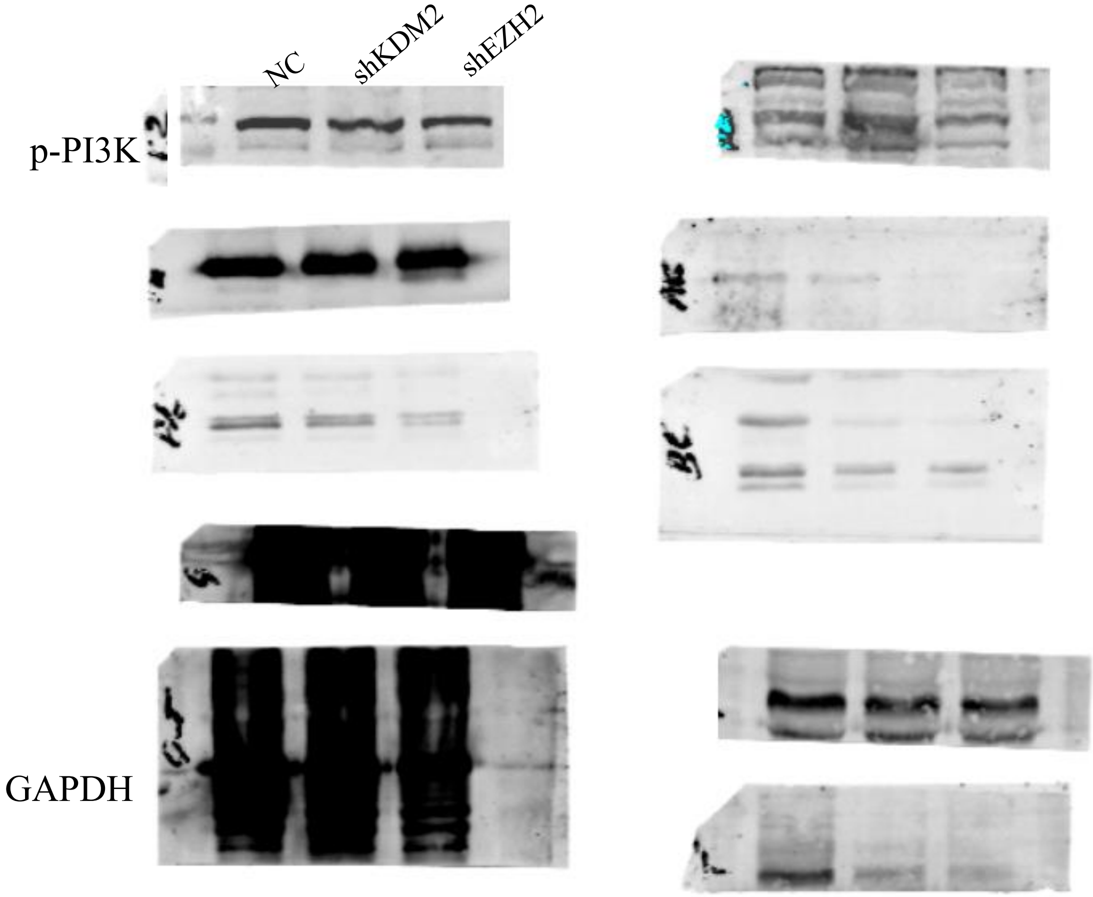

NC
shKDM2
shEZH2
p-PI3K
GAPDH

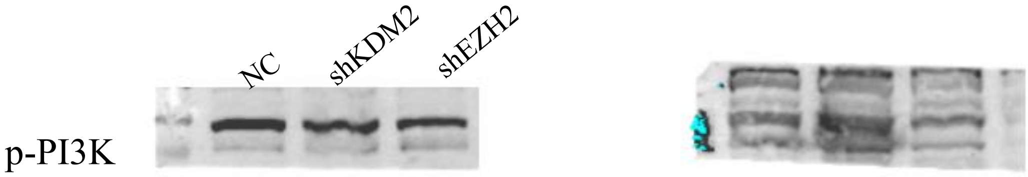

NC
shKDM2
shEZH2
p-PI3K

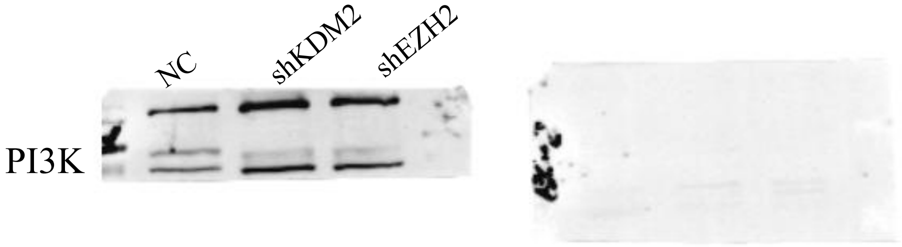

NC
shKDM2
shEZH2
PI3K

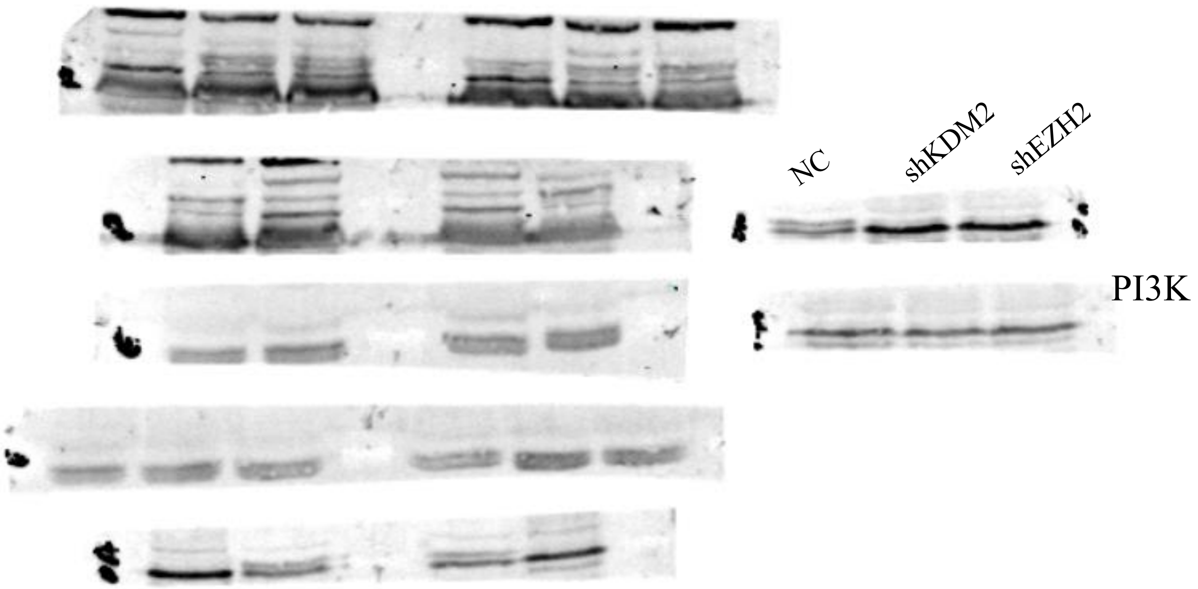
NC
shKDM2
shEZH2
PI3K

9. Protein expression levels of KDM2B and EZH2 in CD133^{-}/CD144^{-} and CD133^{+}/CD144^{+} cells population (**Figure 5**).

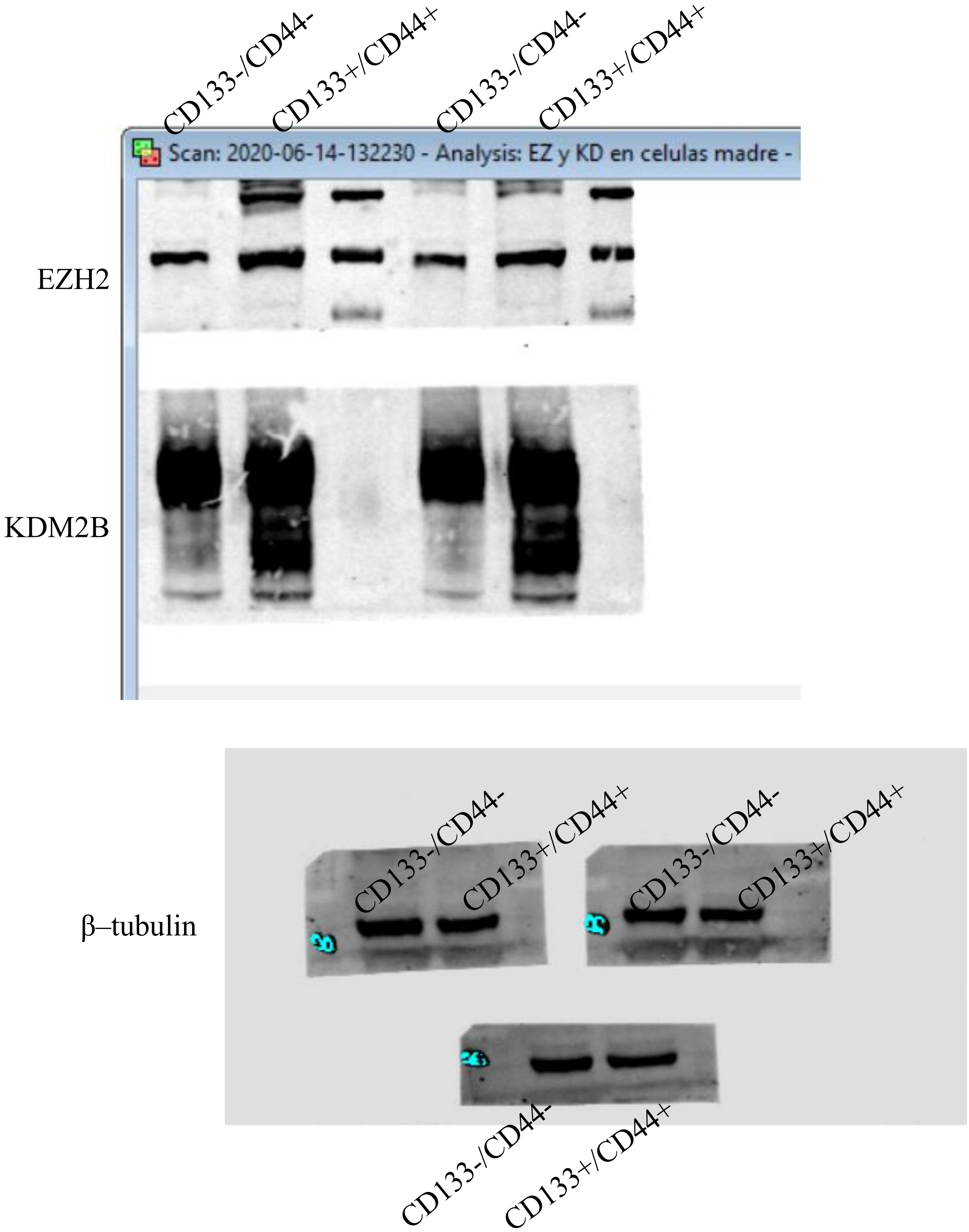

10. The expression of p-PI3K, PI3K, p-AKT, AKT, KDM2B, EZH2, and the stem cell markers CD44, CD133, and ALDH-1 after KDM2B and EZH2 knockdown in CD133 $^{+}$/CD44 $^{+}$ cells population. β-tubulin was used as a loading control (**Figure 5**).

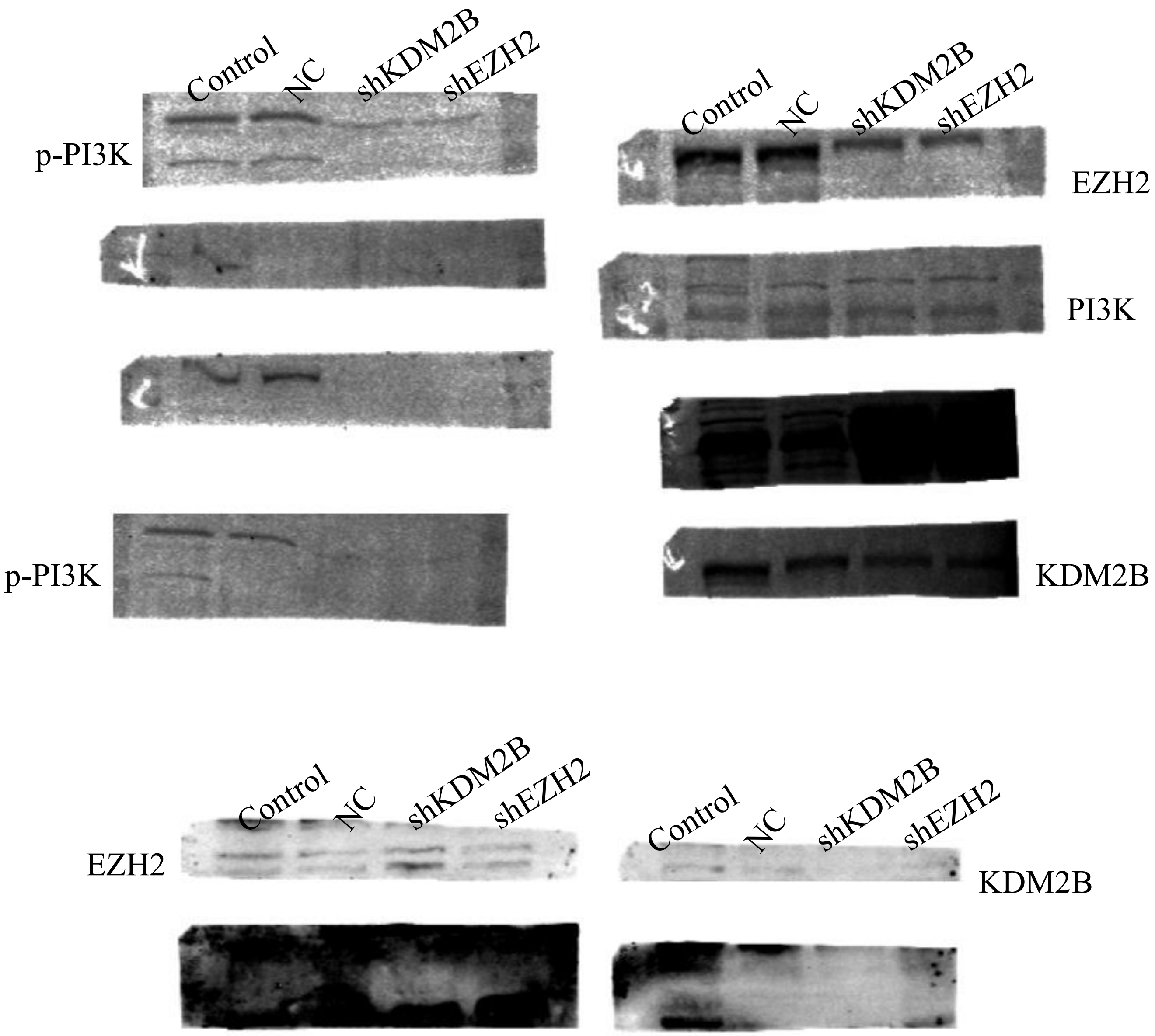

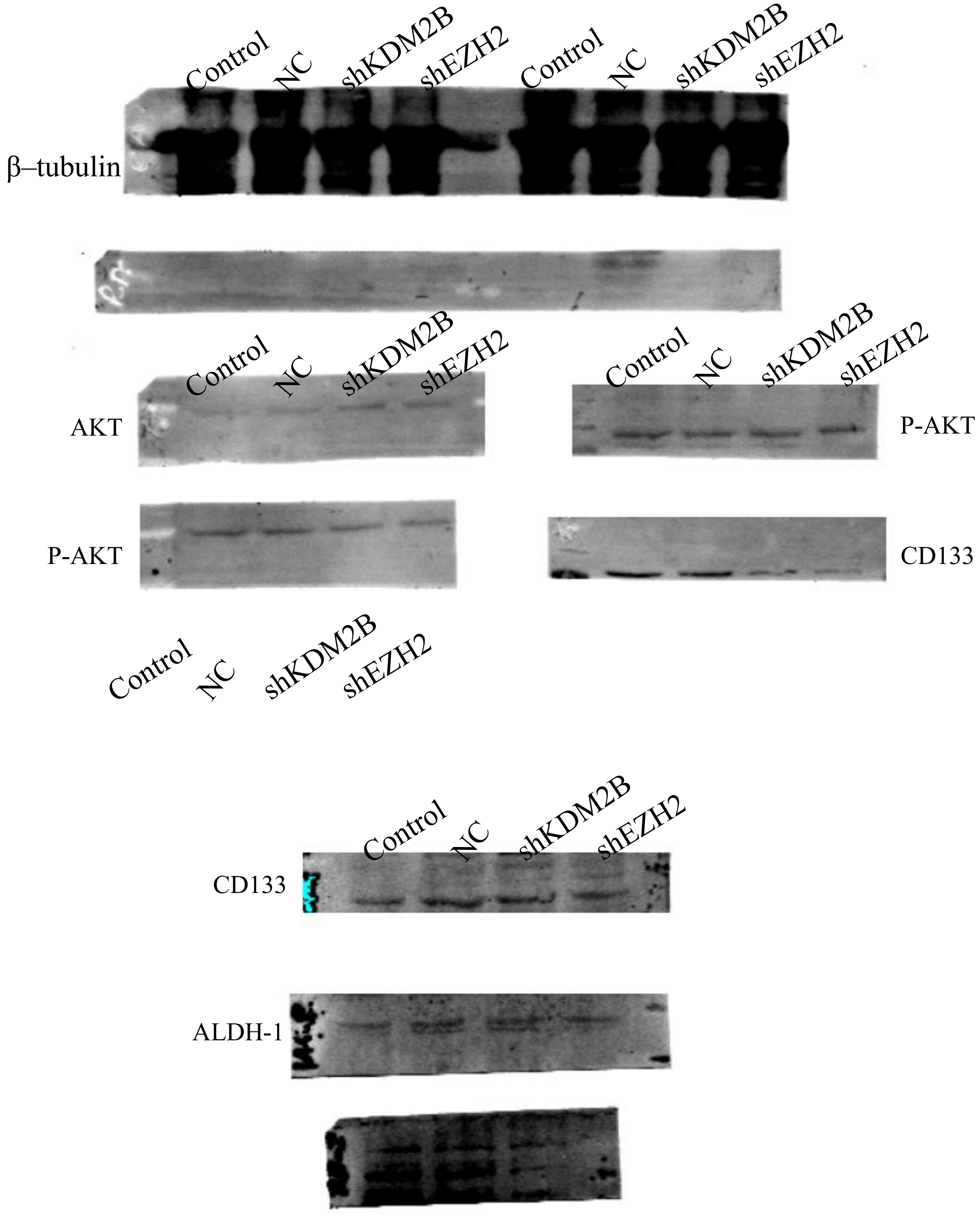
Control
NC
shKDM2B
shEZH2
Control
NC
shKDM2B
shEZH2
β–tubulin
Control
NC
shKDM2B
shEZH2
AKT
Control
NC
shKDM2B
shEZH2
P-AKT
P-AKT
CD133
Control
NC
shKDM2B
shEZH2
Control
NC
shKDM2B
shEZH2
CD133
ALDH-1

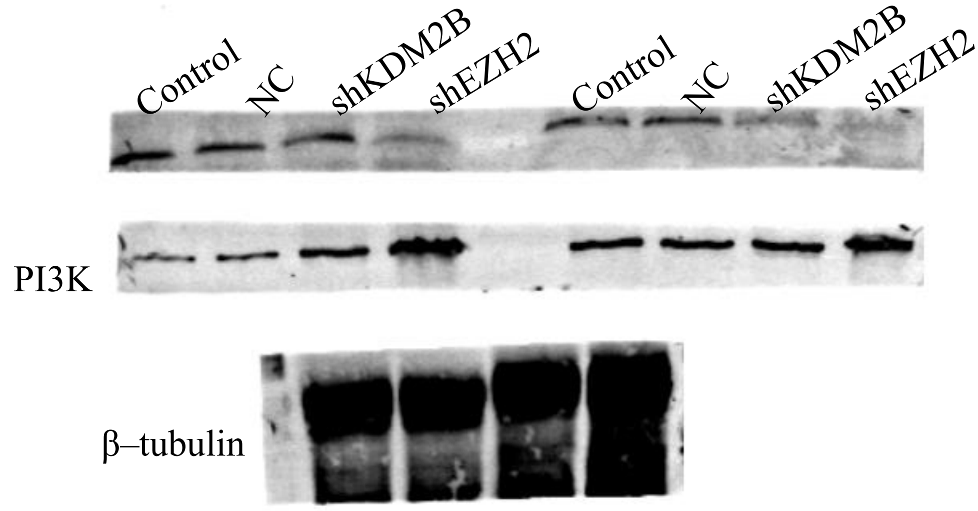
Control
NC
shKDM2B
shEZH2
Control
NC
shKDM2B
shEZH2
PI3K
β–tubulin

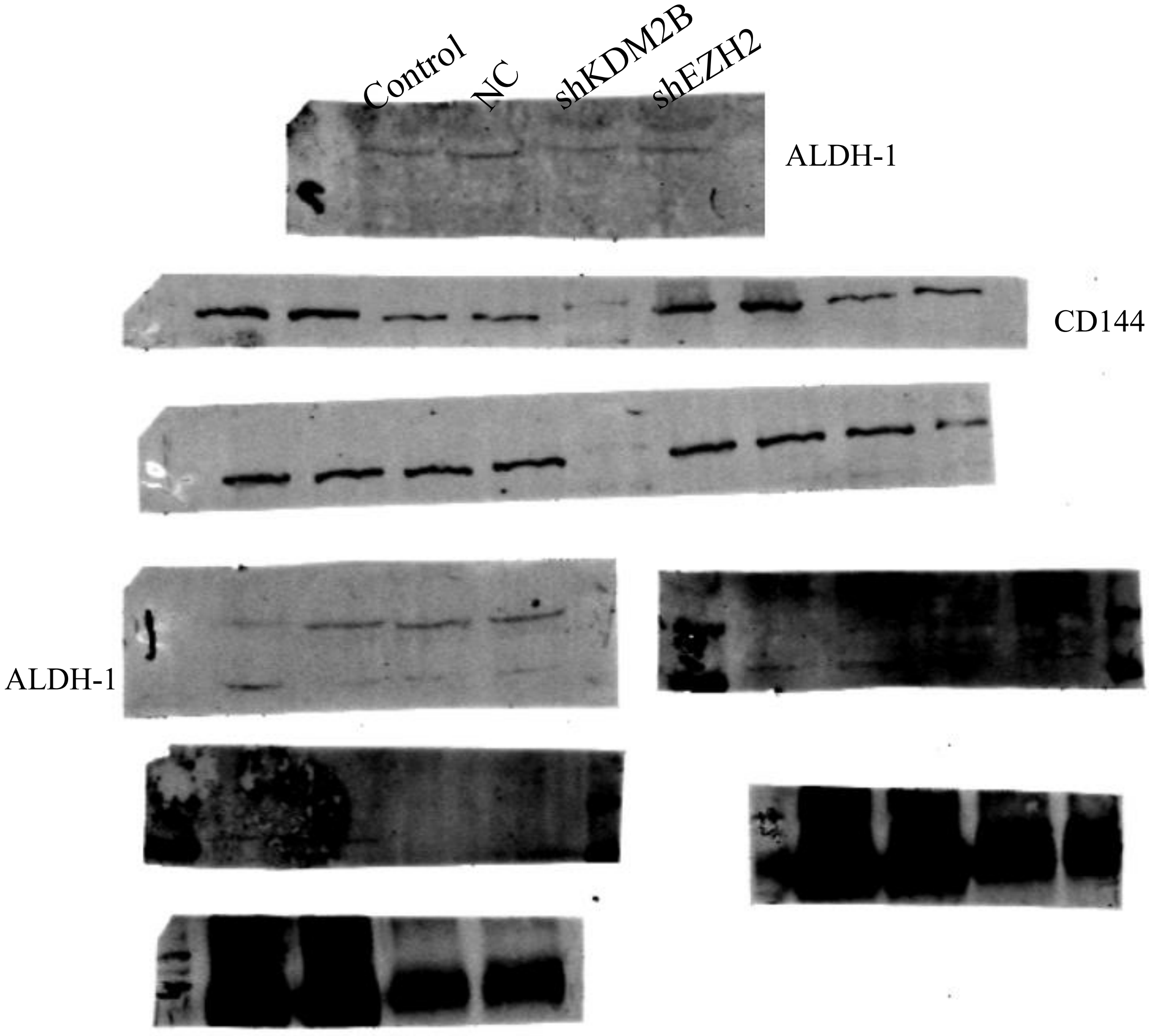
Control
NC
shKDM2B
shEZH2
ALDH-1
CD144
ALDH-1
