# Supplementary material for: The Role of KDM2B and EZH2 in Regulating the Stemness in Colorectal Cancer Through the PI3K/AKT Pathway
Source: Front Oncol. 2021 Mar 9;11:637298. doi: 10.3389/fonc.2021.637298 (PMC8006351; doi:10.3389/fonc.2021.637298)
Supplement: Supplementary file 2 [file Table_1.DOCX]

| ***Primer name*** | ***Primers sequences*** |
| --- | --- |
| **KDM2B** | **F**:5 ′ - CCCAAATGCCTCCTCCACAT -3  **R**: 5 ′ - TCGTTCTCGTCGTATCGCTG-3 |
| **GAPDH** | **F**: 5'- GCACCGTCAAGGCTGAGAAC-3'  **R**: 5'-TGGTGAAGACGCCAGTGGA-3' |

**Supplementary Table 1 The Primers sequences of KDM2B and GAPDH**
